# Supplementary material for: New Substituted Benzoylthiourea Derivatives: From Design to Antimicrobial Applications
Source: Molecules. 2020 Mar 25;25(7):1478. doi: 10.3390/molecules25071478 (PMC7180980; doi:10.3390/molecules25071478)
Supplement: Supplementary file 1 [file molecules-25-01478-s001.pdf]

molecules

MDPI

Supplementary materials

New Substituted Benzoylthiourea Derivatives: From Design to Antimicrobial Applications

Carmen Limban [1], Mariana Carmen Chifiriuc [2,*], Miron Teodor Caproiu [3], Florea Dumitrascu [3], Marilena Ferbinteanu [4], Lucia Pintilie [5], Amalia Stefaniu [5], Ilinca Margareta Vlad [1], Coralia Bleotu [6], Luminita Gabriela Marutescu [2] and Diana Camelia Nuta [1]

[1] Department of Pharmaceutical Chemistry, "Carol Davila" University of Medicine and Pharmacy, 020,956 Bucharest, Romania; carmen.limban@umfcd.ro (C.L.); ilinca.vlad@drd.umfcd.ro (I.M.V.); diana.nuta@umfcd.ro (D.C.N.)

[2] Department of Microbiology, Faculty of Biology & Research Institute of the University of Bucharest (ICUB), University of Bucharest, 060,101 Bucharest, Romania; lumi.marutescu@gmail.com

[3] The Organic Chemistry Center of Romanian Academy "C. D. Neniţescu", 060,023 Bucharest, Romania; dorucaproiu@gmail.com (M.T.C.); fdumitra@yahoo.com (F.D.)

[4] Inorganic Chemistry Department, Faculty of Chemistry, University of Bucharest, 020,462 Bucharest, Romania; marilena.cimpoesu@g.unibuc.ro

[5] National Institute of Chemical-Pharmaceutical Research & Development, 031,299 Bucharest, Romania; lucia.pintilie@gmail.com (L.P.); astefaniu@gmail.com (A.S.)

[6] Stefan S. Nicolau Institute of Virology, Mihai Bravu 285, Bucharest, 030304, Romania; cbleotu@yahoo.com

* Correspondence: carmen.chifiriuc@bio.unibuc.ro

Received: 21 February 2020; Accepted:20 March 2020; Published: date

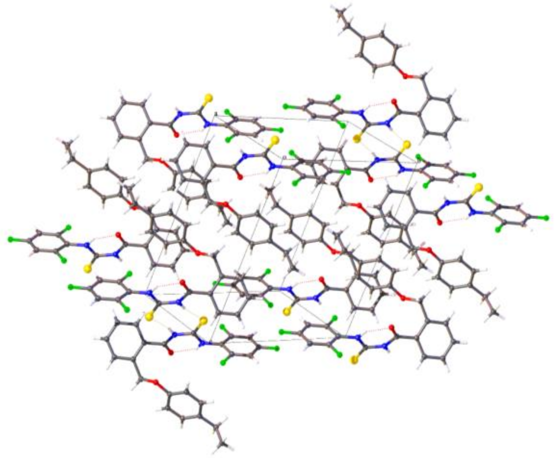

Figure S1. Packing diagram of the crystal structure of **5d** along *a* axis.

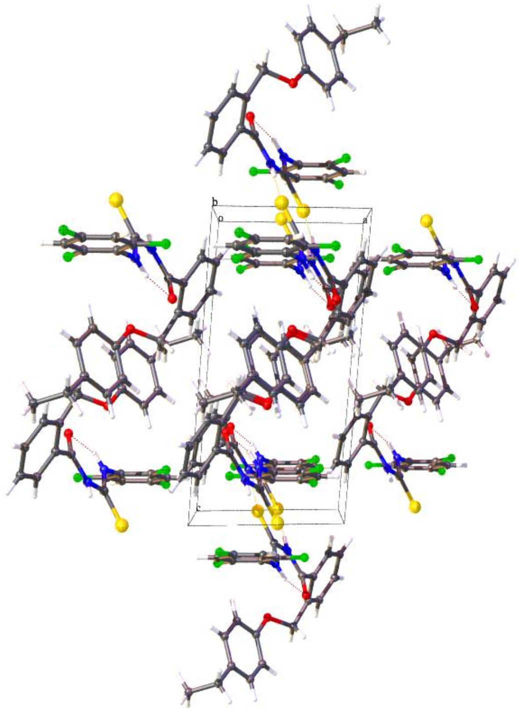

Figure S2. Packing diagram of the crystal structure of **5d** along *b* axis.

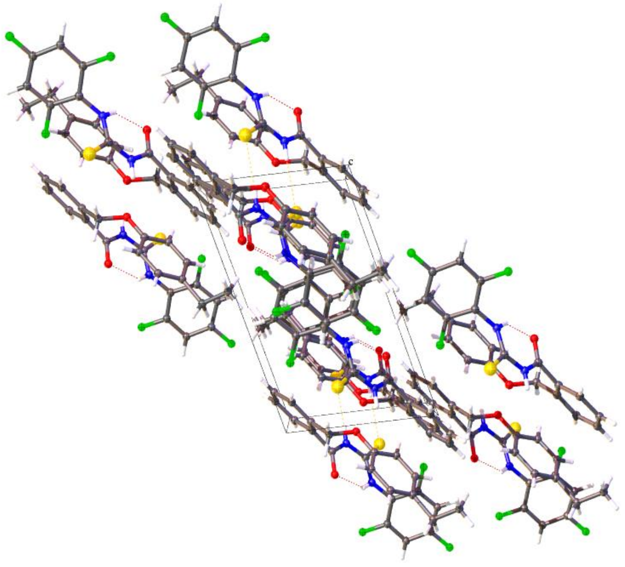

Figure S3. Packing diagram of the crystal structure of **5d** along *c* axis.

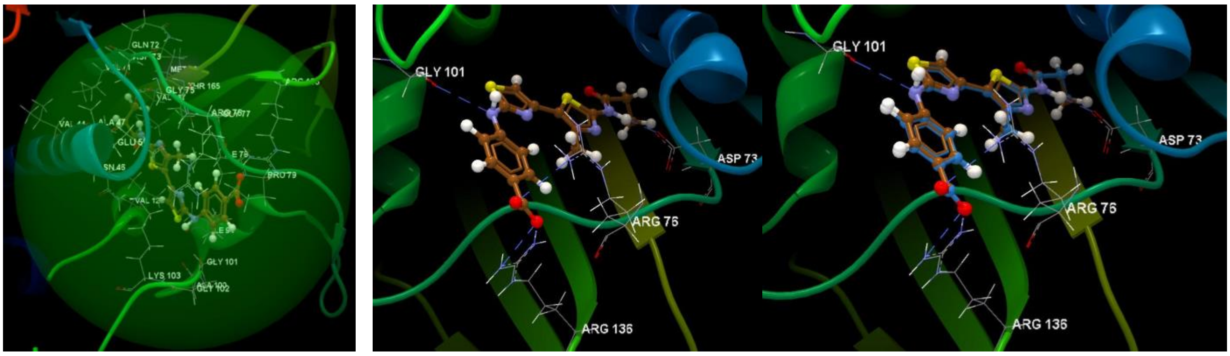

(**a**) Binding site and docking pose of the co-crystallized ligand interacting with residues.

(**b**) Hydrogen bonds between the residues of the ARG 136, ARG 76, GLT 101 and ASP 73 and the co-crystallized

(**c**) Docking validation of co-crystalized ligand

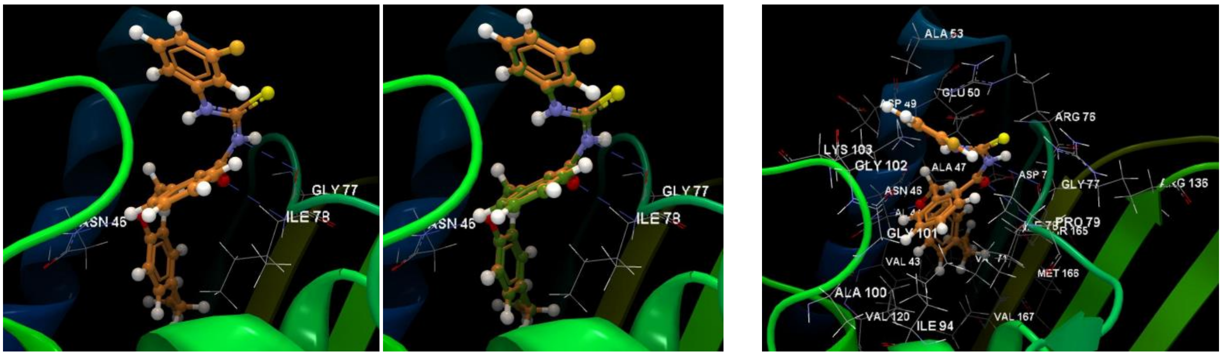

(**d**) Hydrogen bonds between the residues of the ASN 46, ILE 78, GLY 77 and the **5a** ligand

(**e**) Docking validation of **5a.**

(**f**) Docking pose of the **5a** interacting with residues in the binding site.

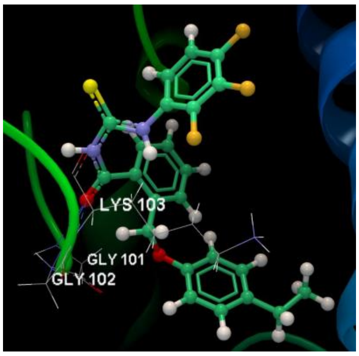

(**g**). Hydrogen bonds between the residues of the LYS 103, GLY 102, GLY101 and the **5b** ligand.

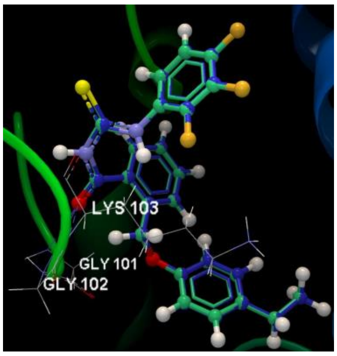

(**h**) Docking validation of **5b.**

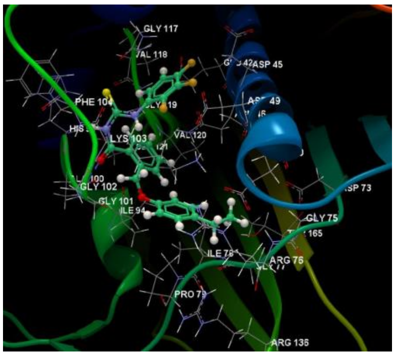

(**i**) Docking pose of the **5b** interacting with residues in the binding site.

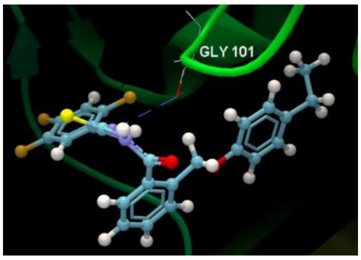

(**j**) Hydrogen bonds between the residues of the GLY 101 and the **5c** ligand.

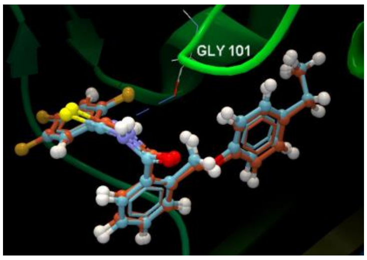

(**k**) Docking validation of **5c.**

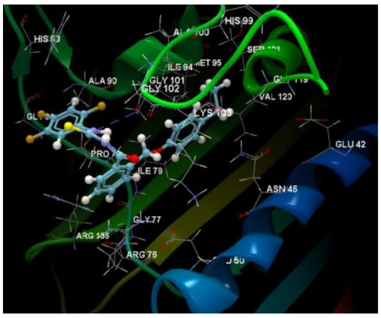

(**l**) Docking pose of the **5c** interacting with residues in the binding site.

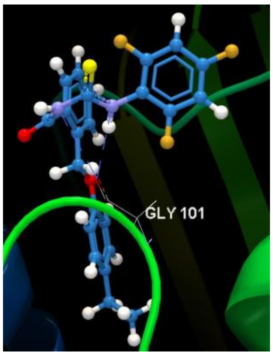

(**m**) Hydrogen bonds between the residues of the GLY 101 and the **5d** ligand.

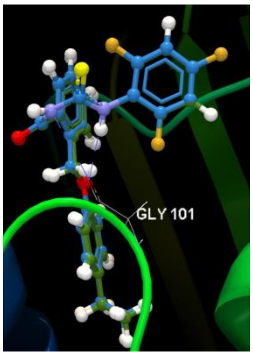

(**n**) Docking validation of **5d**

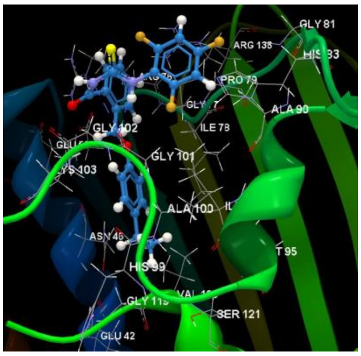

(**o**) Docking pose of the **5d** interacting with residues in the binding site.

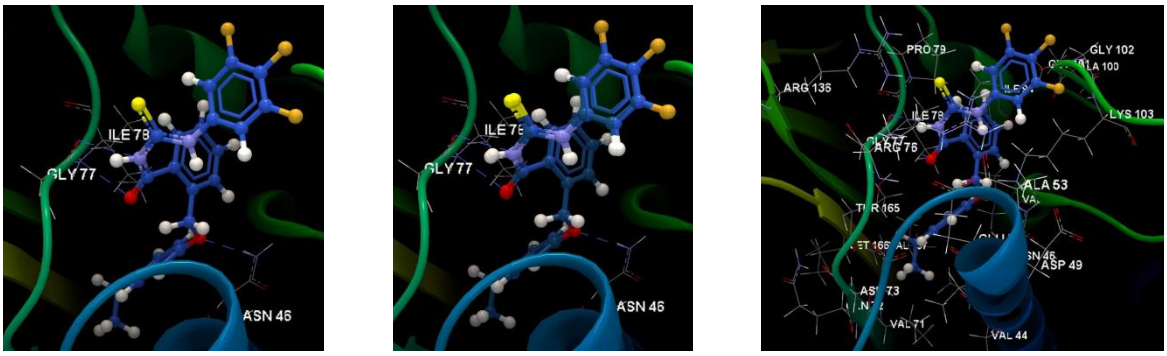

(**p**) Hydrogen bonds between the residues of the ASN 46, ILE 78, GLY 77 and the **5e** ligand.

(**r**) Docking validation of **5e.**

(**s**) Docking pose of the **5e** interacting with residues in the binding site.

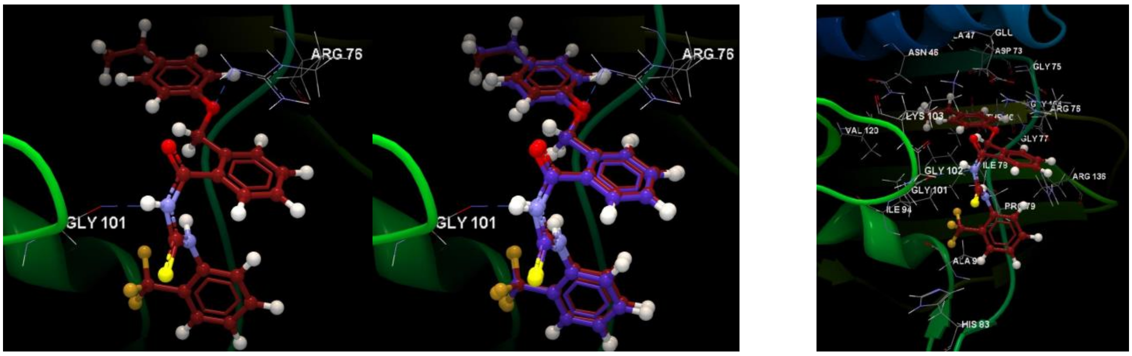

(**t**) Hydrogen bonds between the residues of the GLY 101 si ARG 76 and the **5f** ligand.

(**u**) Docking validation of **5f.**

(**v**) Docking pose of the **5f** interacting with residues in the binding site.

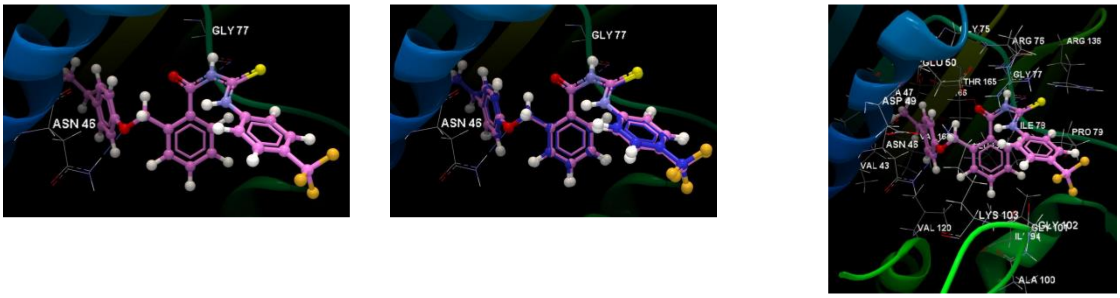

(**x**) Hydrogen bonds between the residues of the ASN 46, GLY 77 and the **5g** ligand.

(**y**) Docking validation of **5g.**

(**z**) Docking pose of the **5g** interacting with residues in the binding site.

Figure S4. Molecular docking studies with 4DUH receptor.

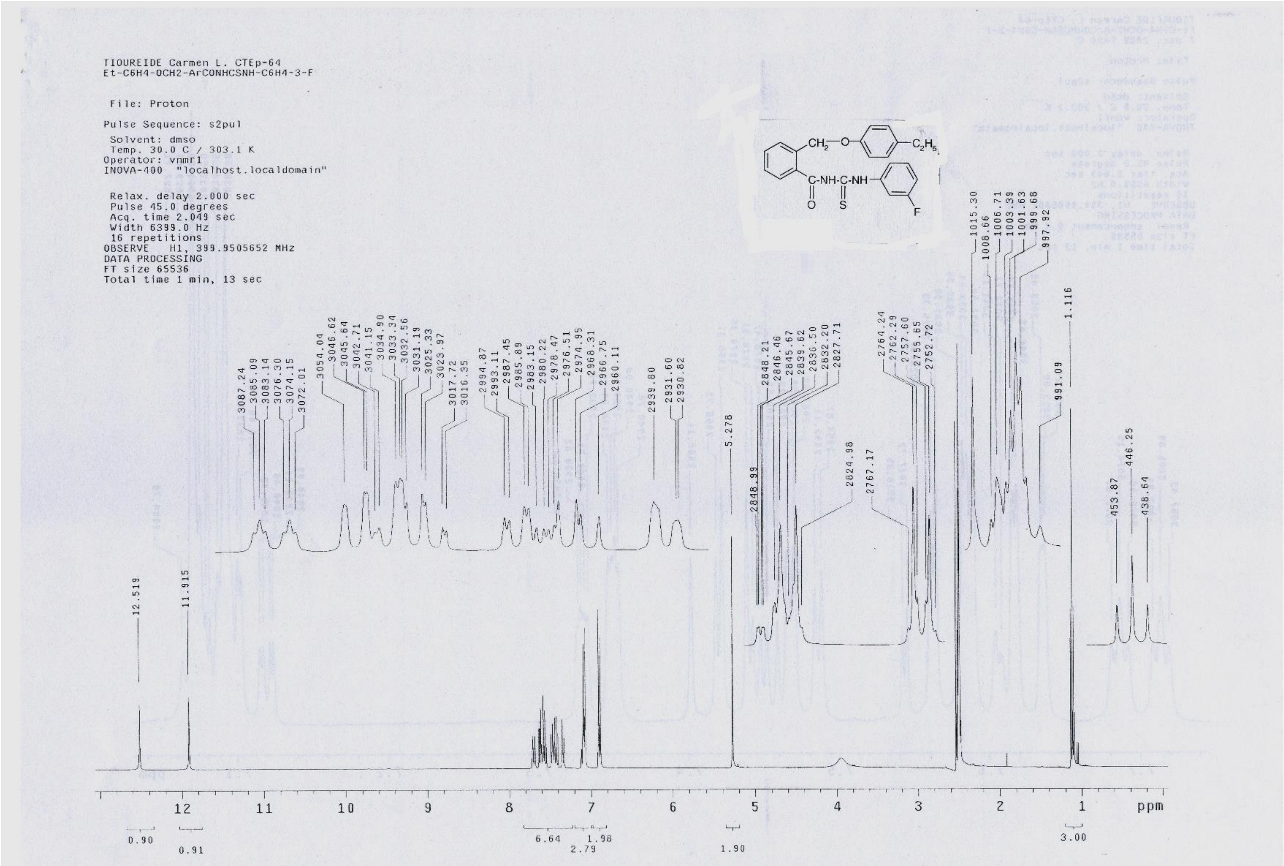

Figure S5. The ^{1}H-NMR spectrum of 2-((4-Ethylphenoxy)methyl)-N-(3-fluorophenylcarbamothioyl)benzamide (**5a**).

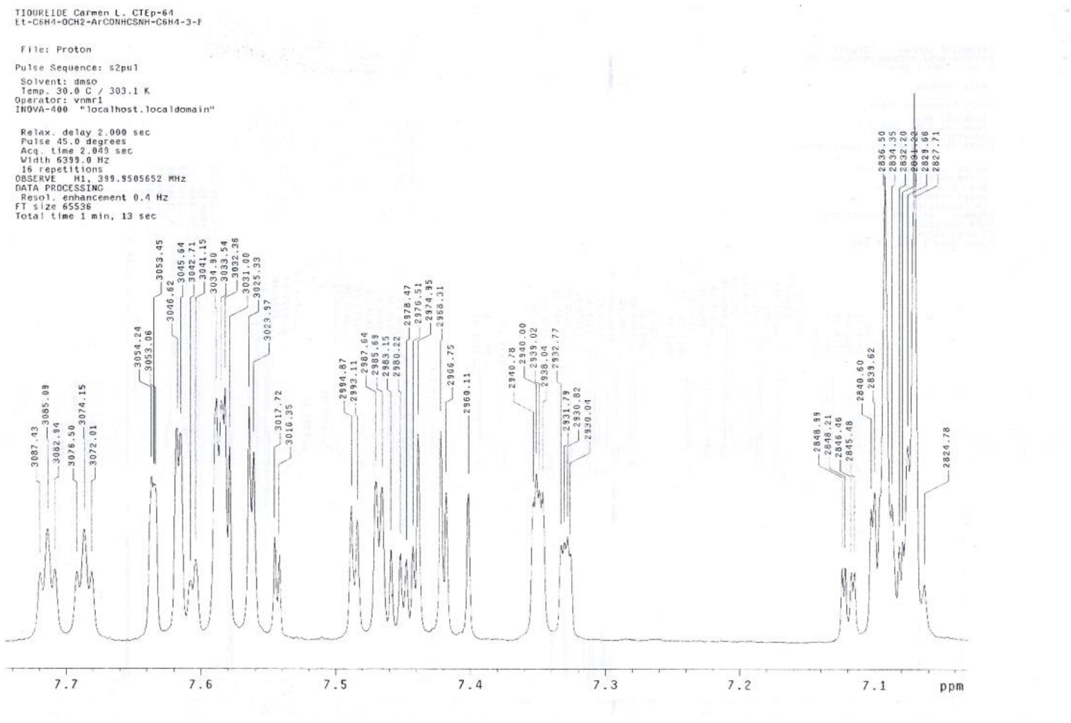

Figure S5. The ^{1}H-NMR spectrum of 2-((4-Ethylphenoxy)methyl)-N-(3-fluorophenylcarbamothioyl)benzamide (**5a**).

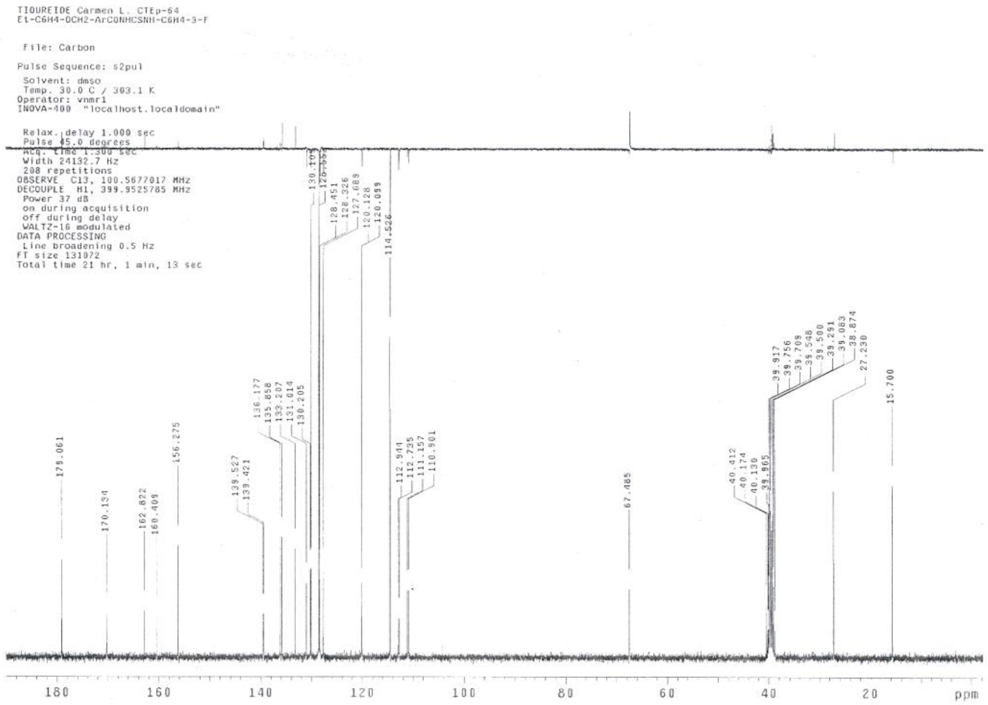

Figure S5. The ^{13}C-NMR spectrum of 2-((4-Ethylphenoxy)methyl)-N-(3-fluorophenylcarbamothioyl)benzamide (**5a**).

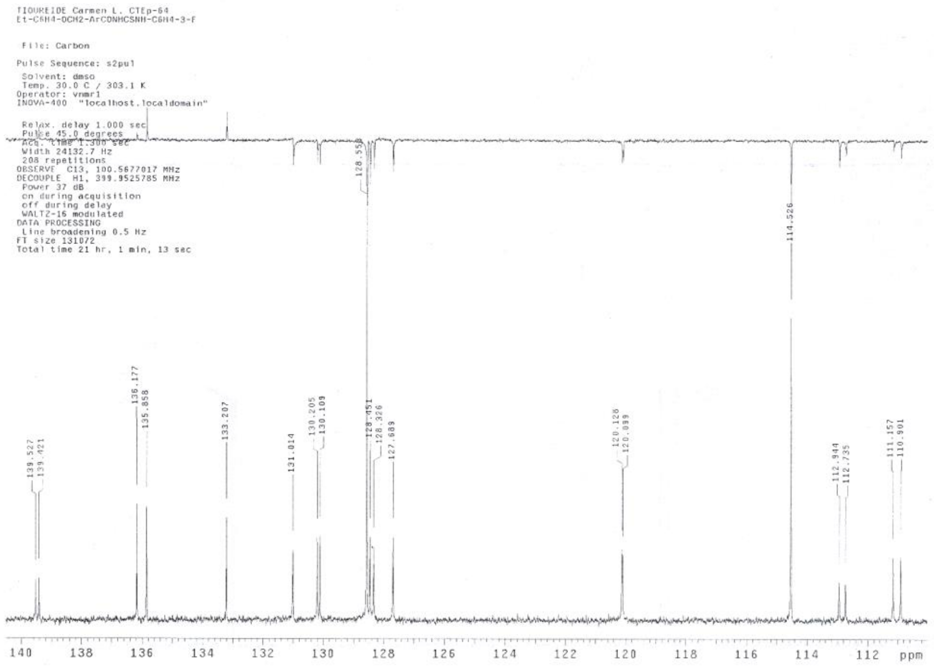

Figure S5. The ^{13}C-NMR spectrum of 2-((4-Ethylphenoxy)methyl)-N-(3-fluorophenylcarbamothioyl)benzamide (**5a**).

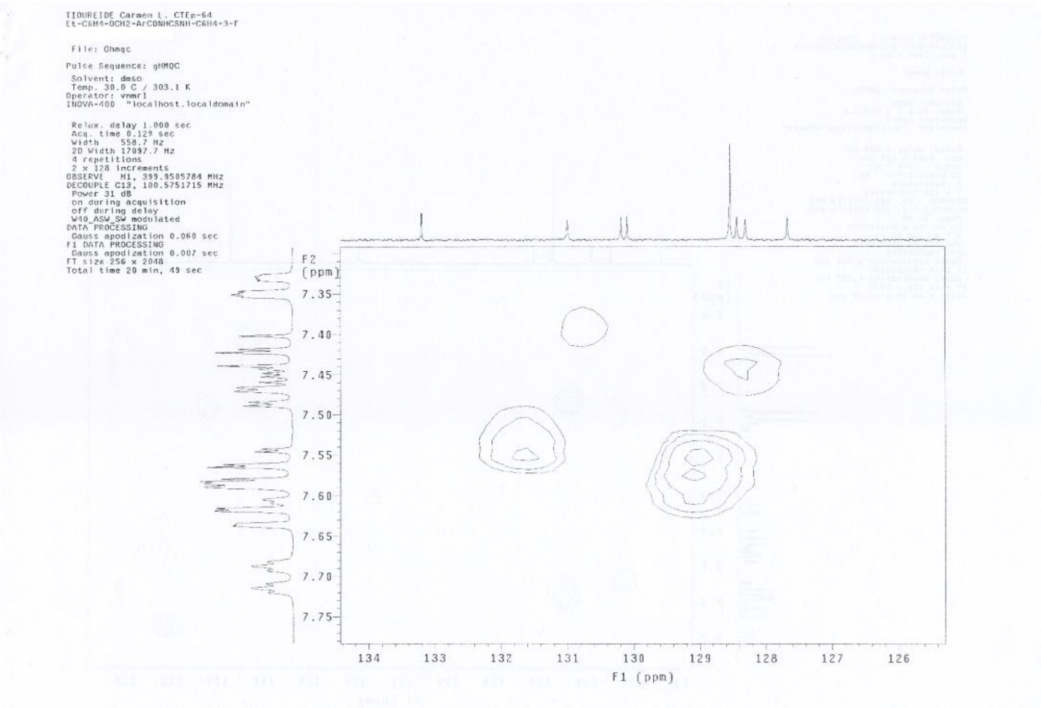

Figure S5. The gHMQC spectrum of 2-((4-Ethylphenoxy)methyl)-N-(3-fluorophenylcarbamothioyl)benzamide (**5a**).

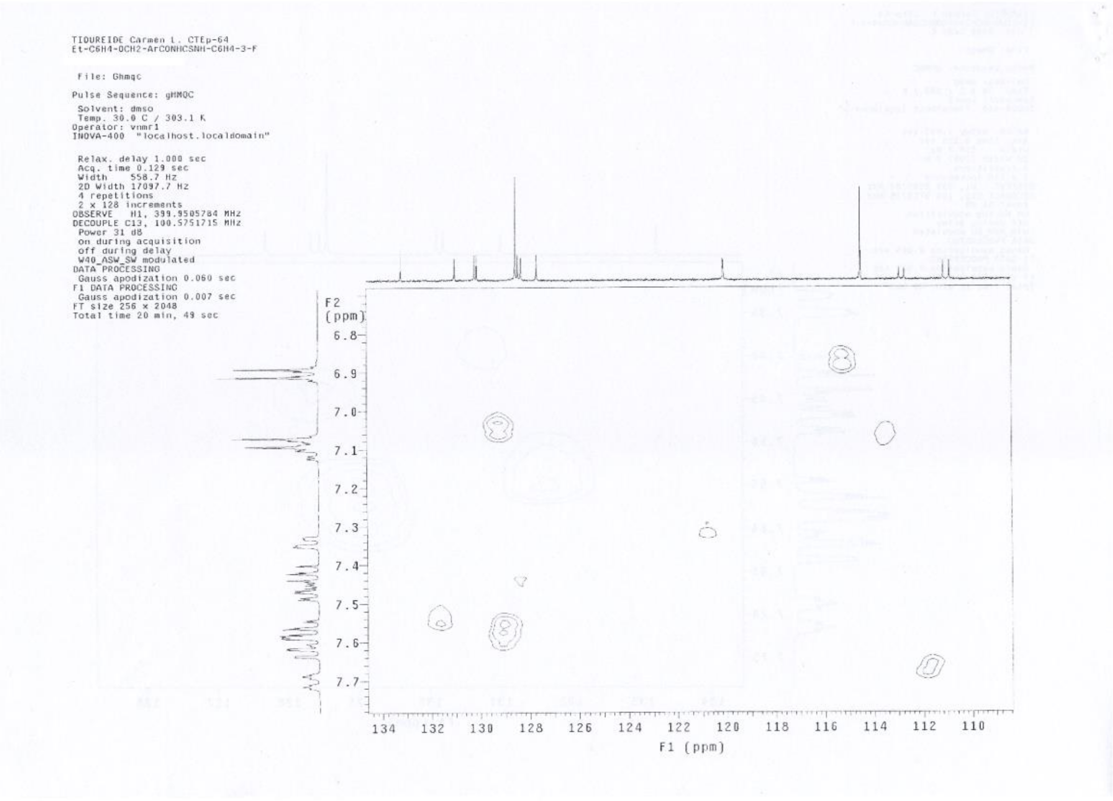

Figure S5. The gHMQC spectrum of 2-((4-Ethylphenoxy)methyl)-N-(3-fluorophenylcarbamothioyl)benzamide (**5a**).

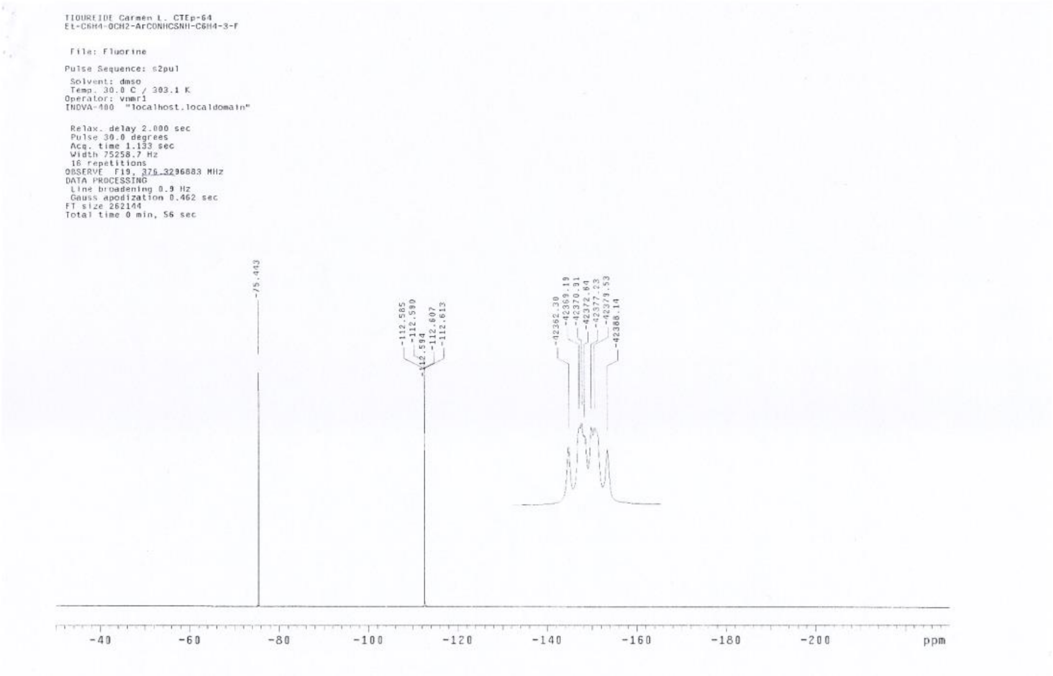

Figure S5. The ^{19}F NMR spectrum of 2-((4-Ethylphenoxy)methyl)-N-(3-fluorophenylcarbamothioyl)benzamide (**5a**).

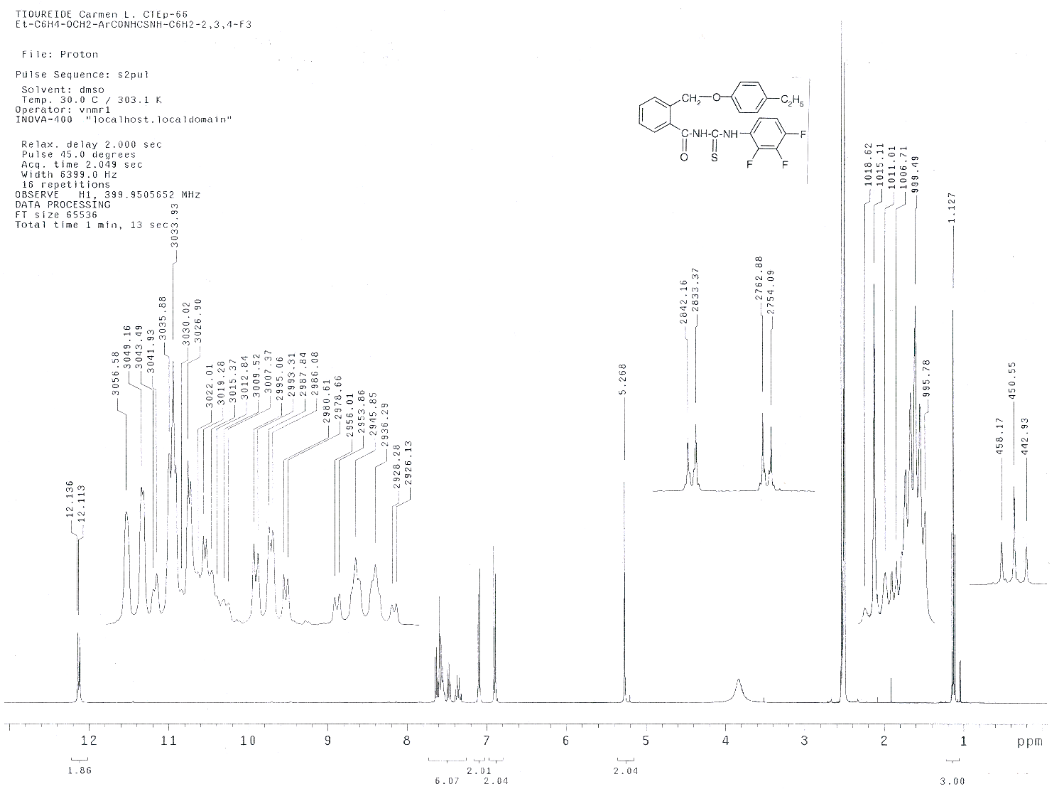

Figure S6. The ^{1}H-NMR spectrum of 2-((4-Ethylphenoxy)methyl)-N-(2,3,4-trifluorophenylcarbamothioyl)benzamide (**5b**).

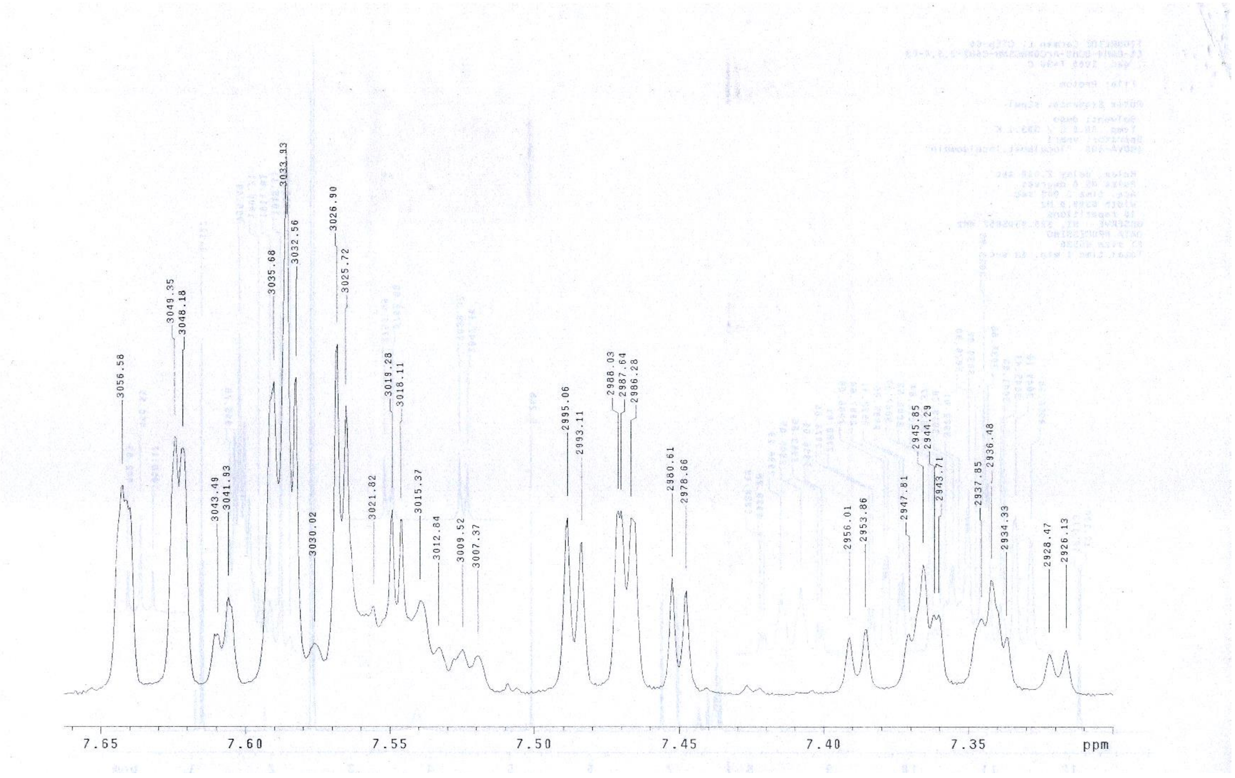

Figure S6. The ^{1}H-NMR spectrum of 2-((4-Ethylphenoxy)methyl)-N-(2,3,4-trifluorophenylcarbamothioyl)benzamide (**5b**).

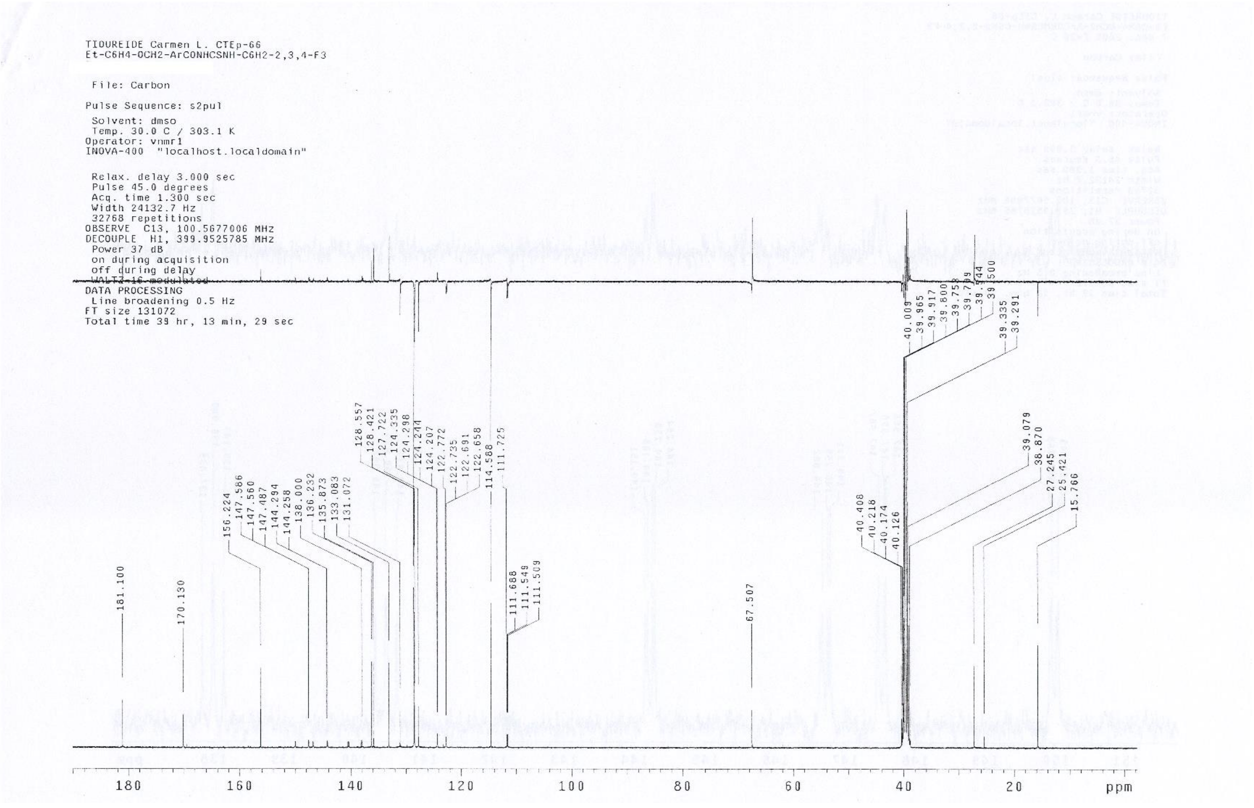

Figure S6. The ^{13}C-NMR spectrum of 2-((4-Ethylphenoxy)methyl)-N-(2,3,4-trifluorophenylcarbamothioyl)benzamide (**5b**).

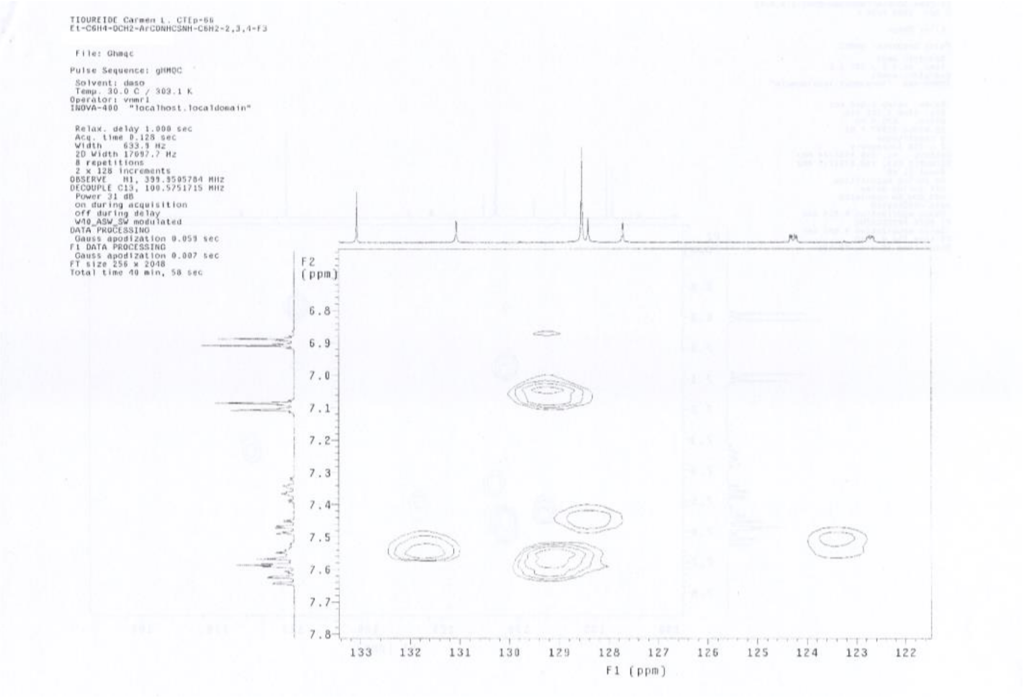

Figure S6. The gHMQC spectrum of 2-((4-Ethylphenoxy)methyl)-N-(2,3,4-trifluorophenylcarbamothioyl)benzamide (**5b**) .

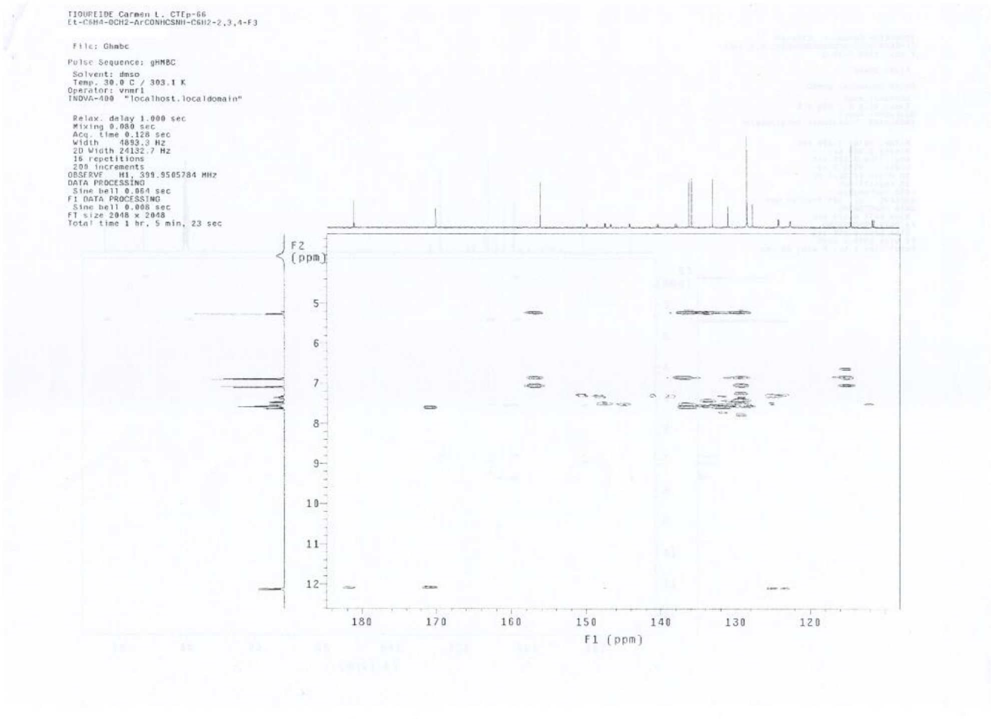

Figure S6. The gHMBC spectrum of 2-((4-Ethylphenoxy)methyl)-N-(2,3,4-trifluorophenylcarbamothioyl)benzamide (**5b**).

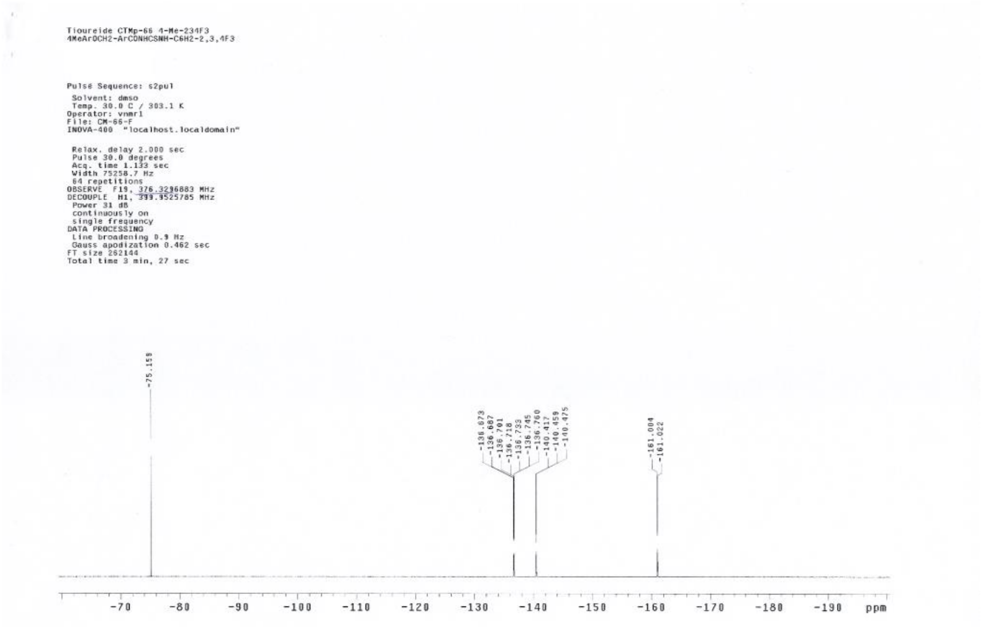

Figure S6. The ^{19}F NMR spectrum of 2-((4-Ethylphenoxy)methyl)-N-(2,3,4-trifluorophenylcarbamothioyl)benzamide (**5b**) .

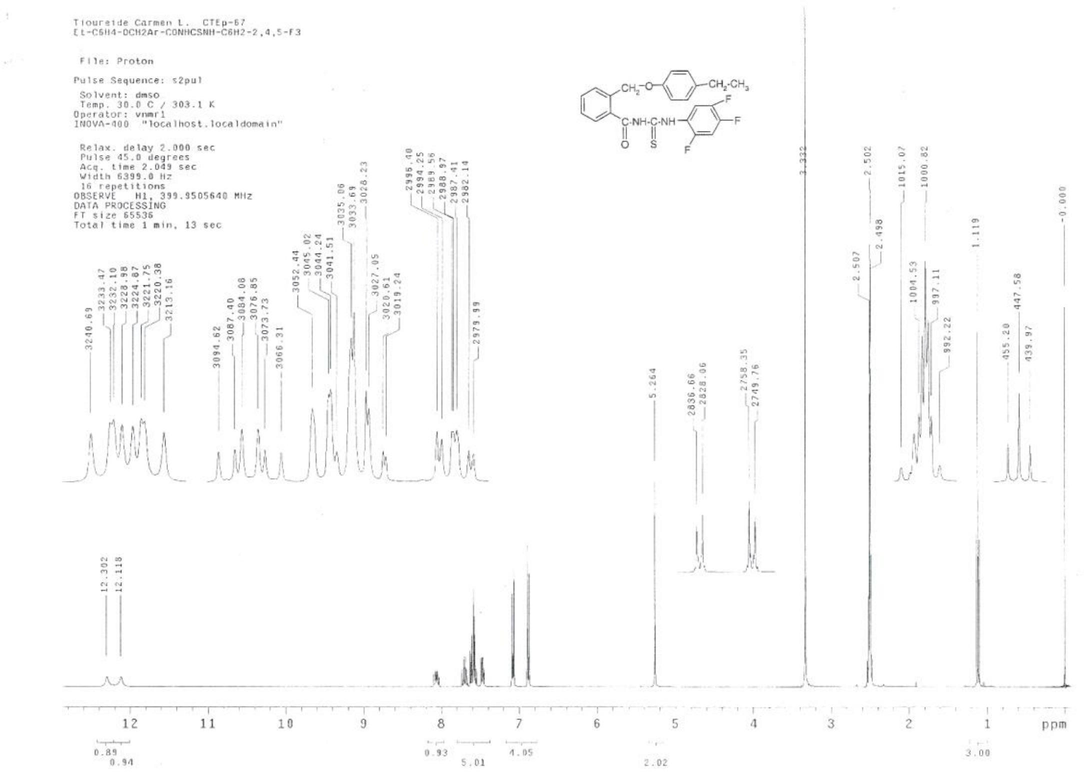

Figure S7. The ^{1}H-NMR spectrum of 2-((4-Ethylphenoxy)methyl)-N-(2,4,5-trifluorophenylcarbamothioyl)benzamide (**5c**).

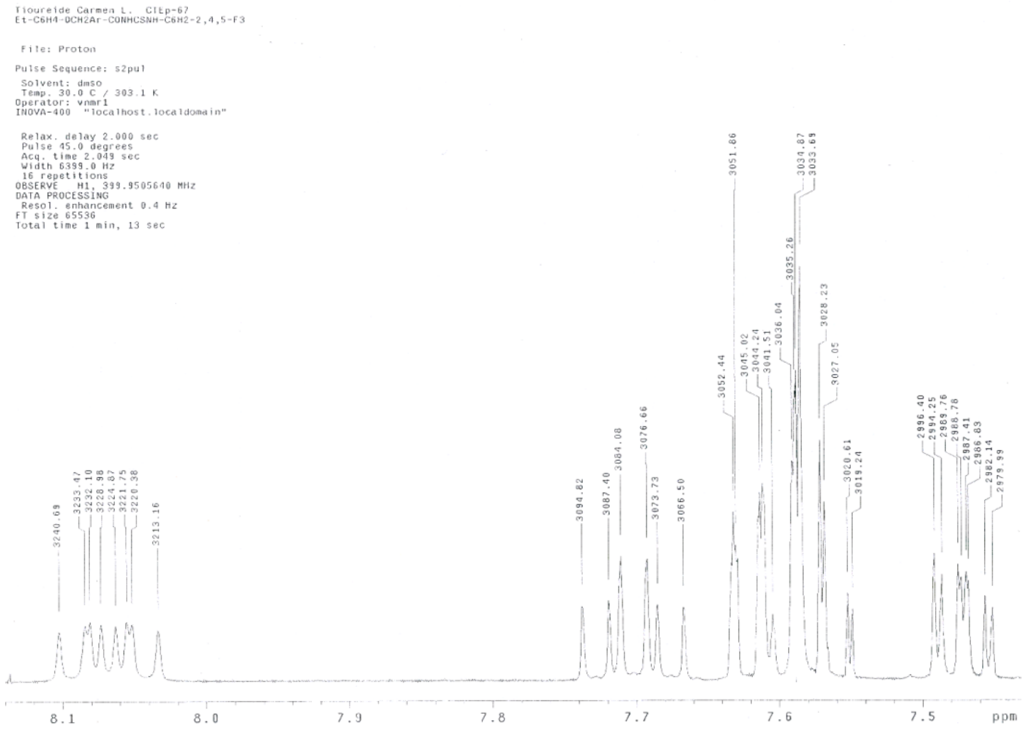

Figure S7. The ^{1}H-NMR spectrum of 2-((4-Ethylphenoxy)methyl)-N-(2,4,5-trifluorophenylcarbamothioyl)benzamide (**5c**).

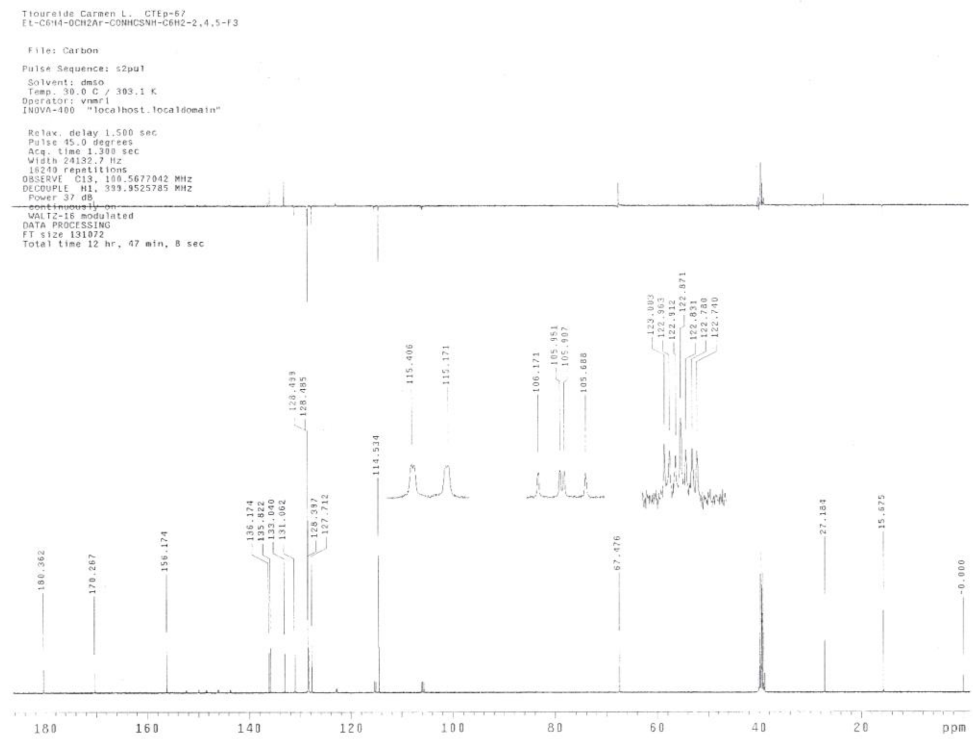

Figure S7. The ^{13}C-NMR spectrum of 2-((4-Ethylphenoxy)methyl)-N-(2,4,5-trifluorophenylcarbamothioyl)benzamide (**5c**).

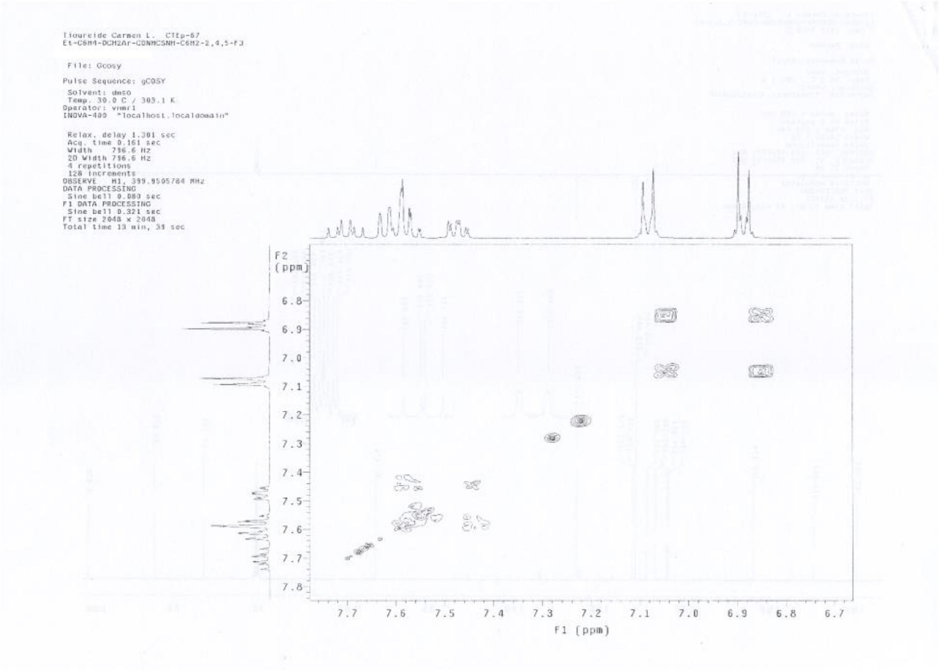

Figure S7. The gCOSY spectrum of 2-((4-Ethylphenoxy)methyl)-N-(2,4,5-trifluorophenylcarbamothioyl)benzamide (**5c**).

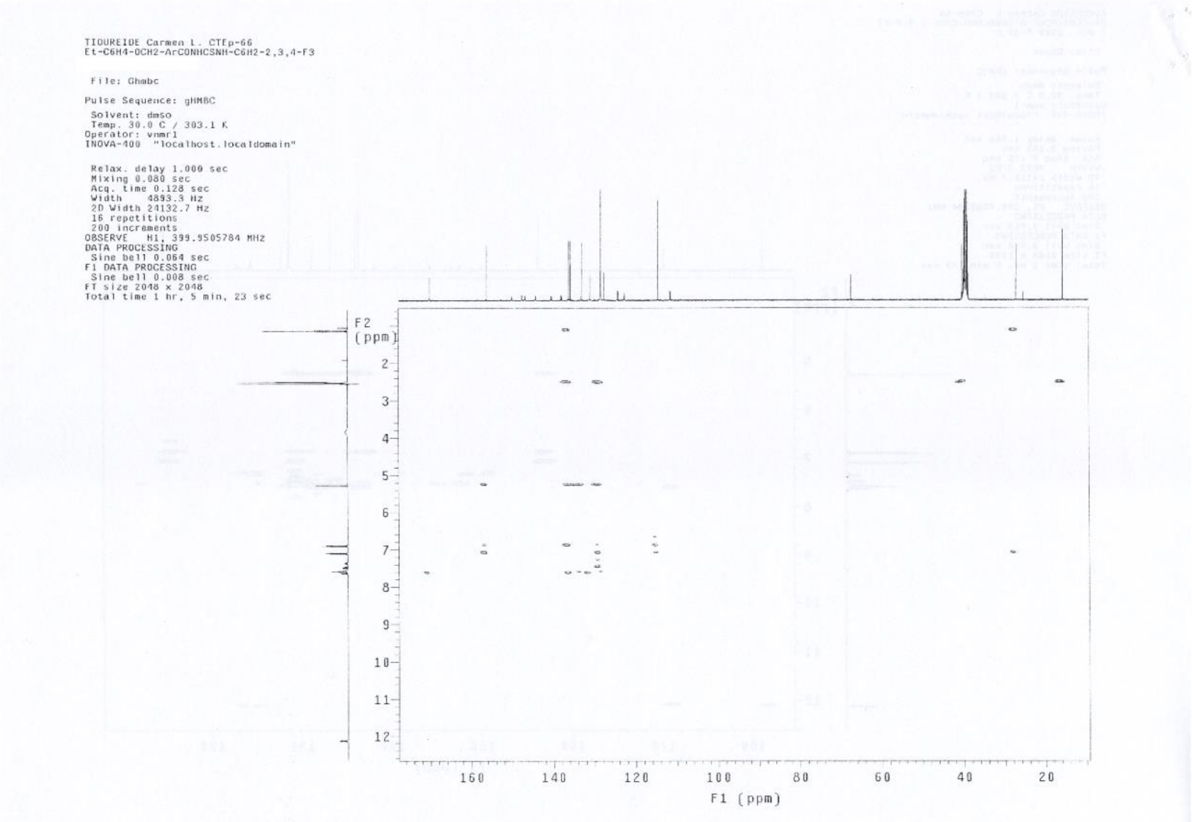

Figure S7. The gHMBC spectrum of 2-((4-Ethylphenoxy)methyl)-N-(2,4,5-trifluorophenylcarbamothioyl)benzamide (**5d**) .

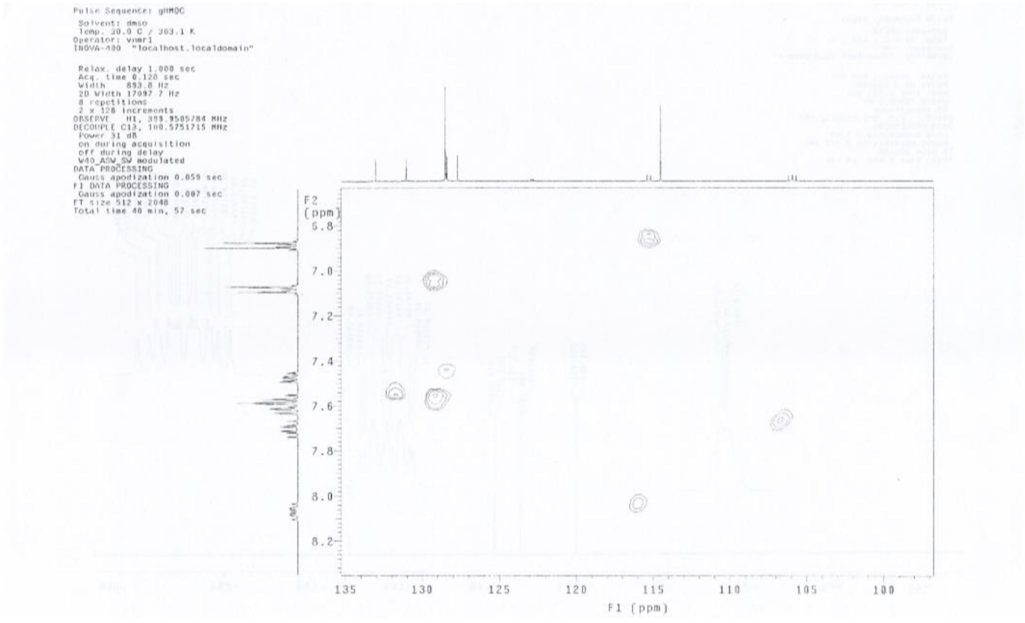

Figure S7. The gHMQC spectrum of 2-((4-Ethylphenoxy)methyl)-N-(2,4,5-trifluorophenylcarbamothioyl)benzamide (**5d**) .

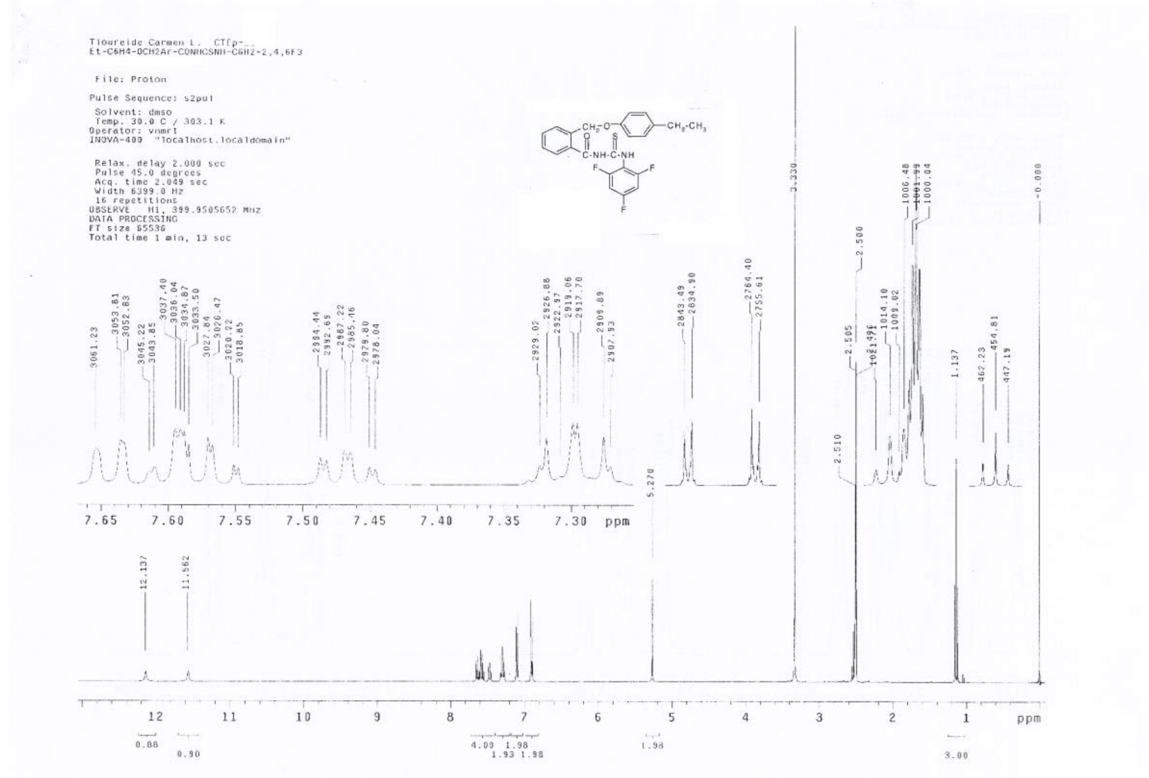

Figure S8. The ^{1}H-NMR spectrum of 2-((4-Ethylphenoxy)methyl)-N-(2,4,6-trifluorophenylcarbamothioyl)benzamide (**5d**).

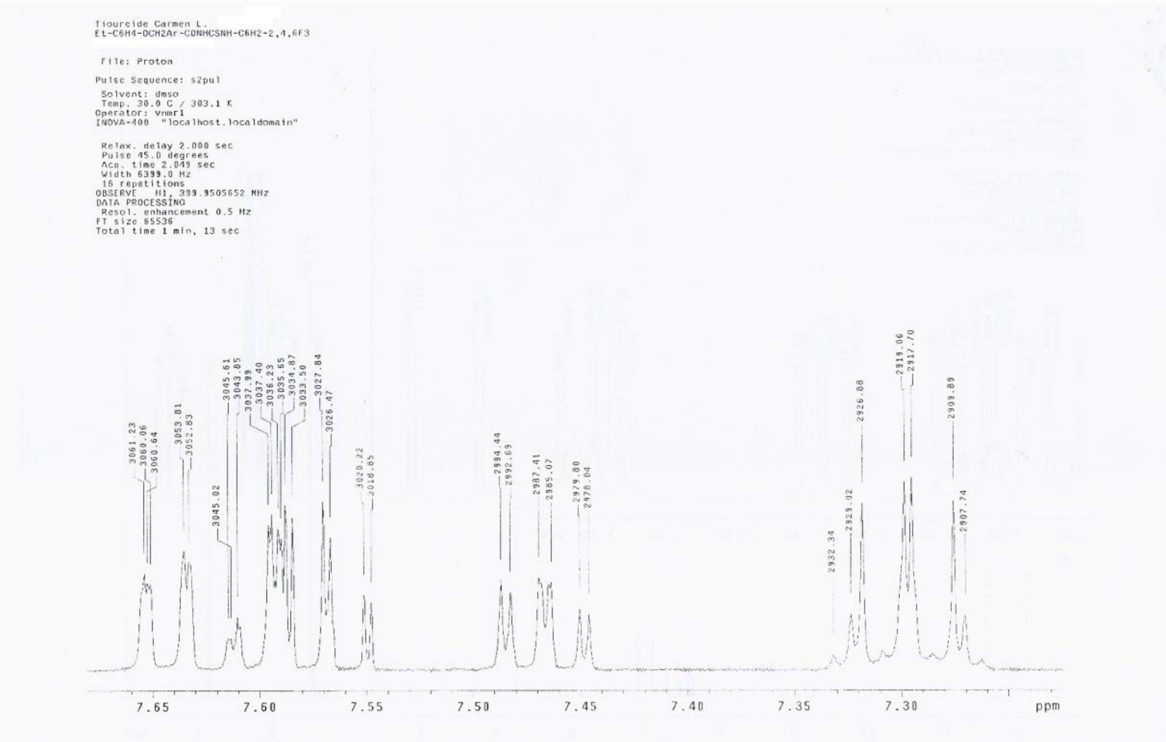

Figure S8. The ^{1}H-NMR spectrum of 2-((4-Ethylphenoxy)methyl)-N-(2,4,6-trifluorophenylcarbamothioyl)benzamide (**5d**).

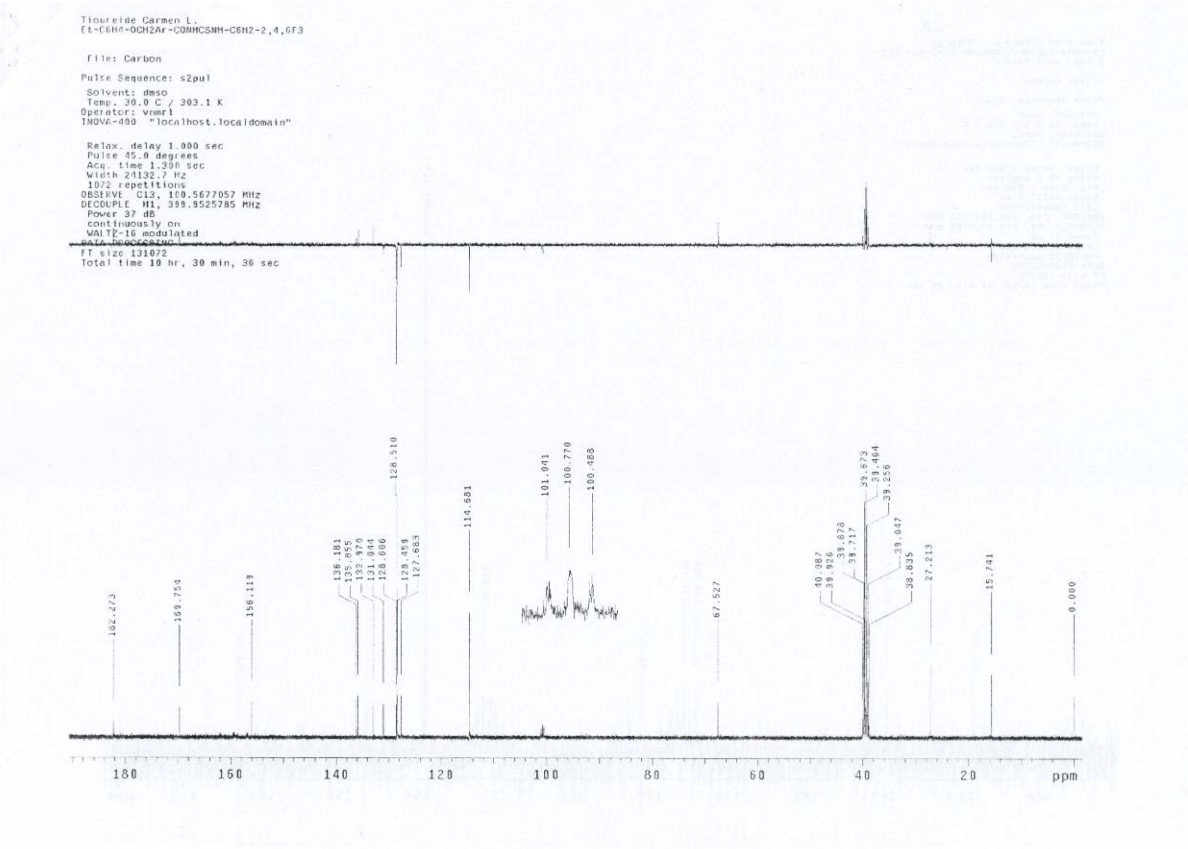

Figure S8. The ^{13}C-NMR spectrum of 2-((4-Ethylphenoxy)methyl)-N-(2,4,6-trifluorophenylcarbamothioyl)benzamide (**5d**).

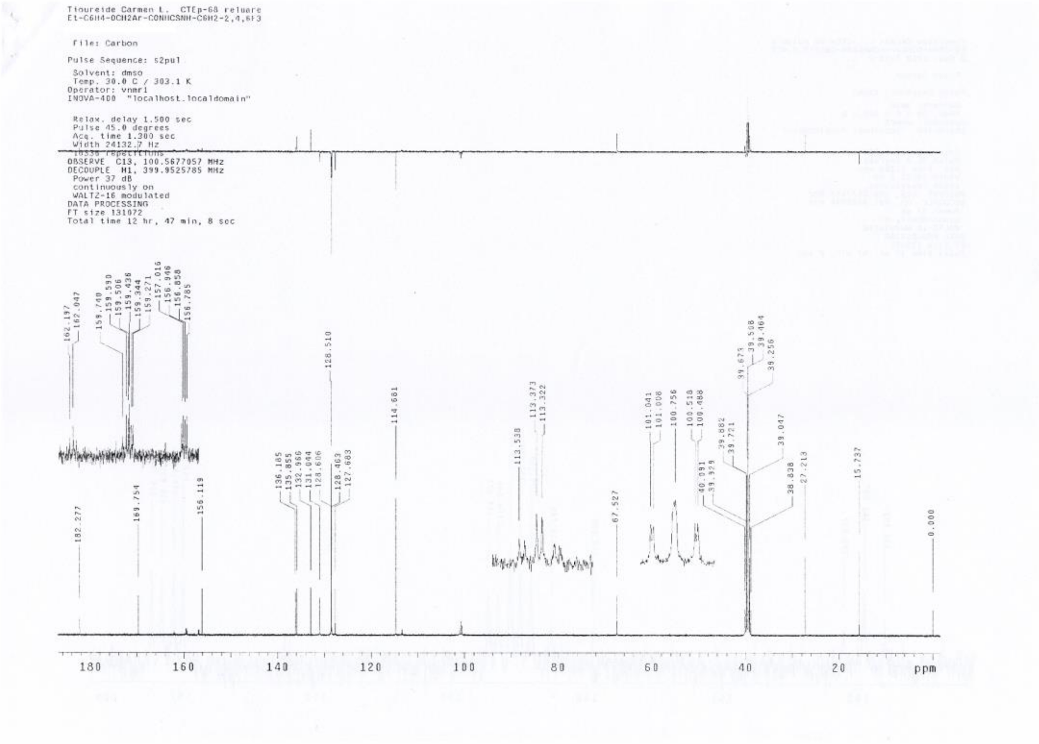

Figure S8. The ^{13}C-NMR spectrum of 2-((4-Ethylphenoxy)methyl)-N-(2,4,6-trifluorophenylcarbamothioyl)benzamide (**5d**).

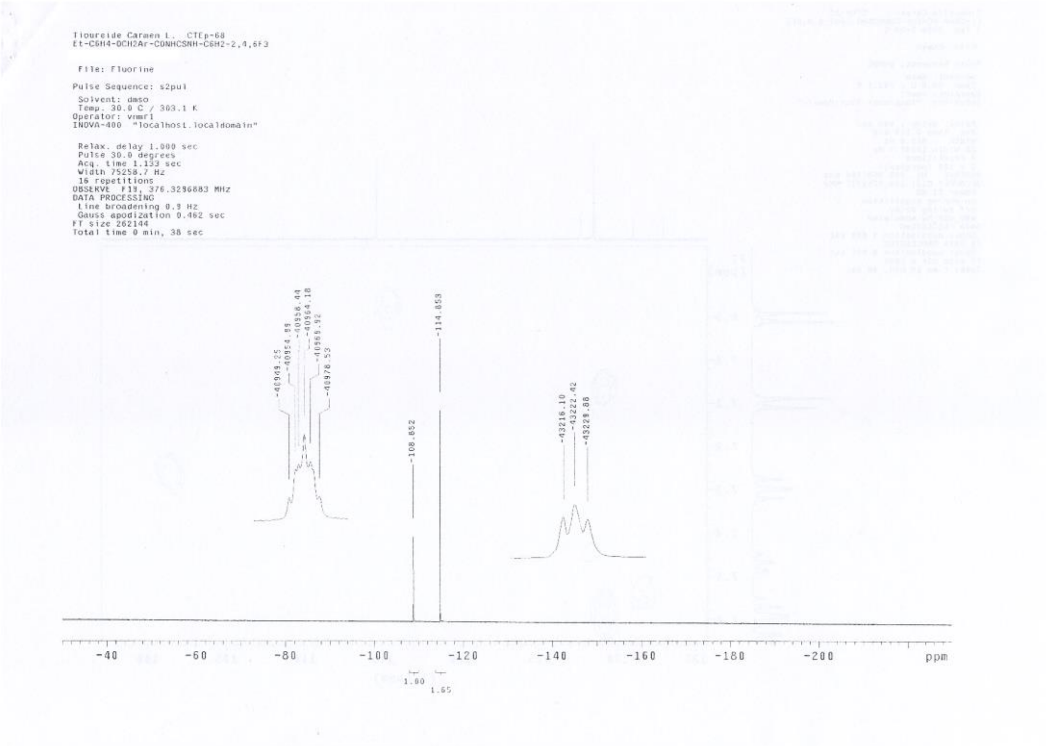

Figure S8. The ^{19}F NMR spectrum of 2-((4-Ethylphenoxy)methyl)-N-(2,4,6-trifluorophenylcarbamothioyl)benzamide (**5d**).

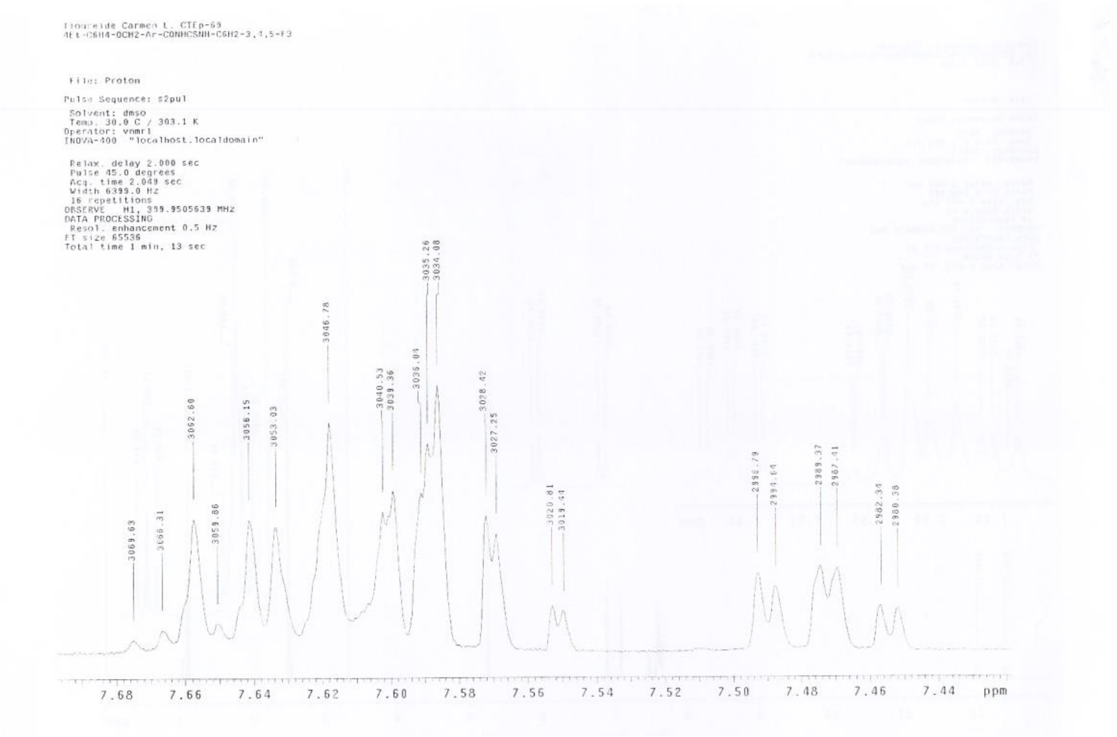

Figure S9. The ^{1}H-NMR spectrum of 2-((4-Ethylphenoxy)methyl)-N-(3,4,5-trifluorophenylcarbamothioyl)benzamide (**5e**).

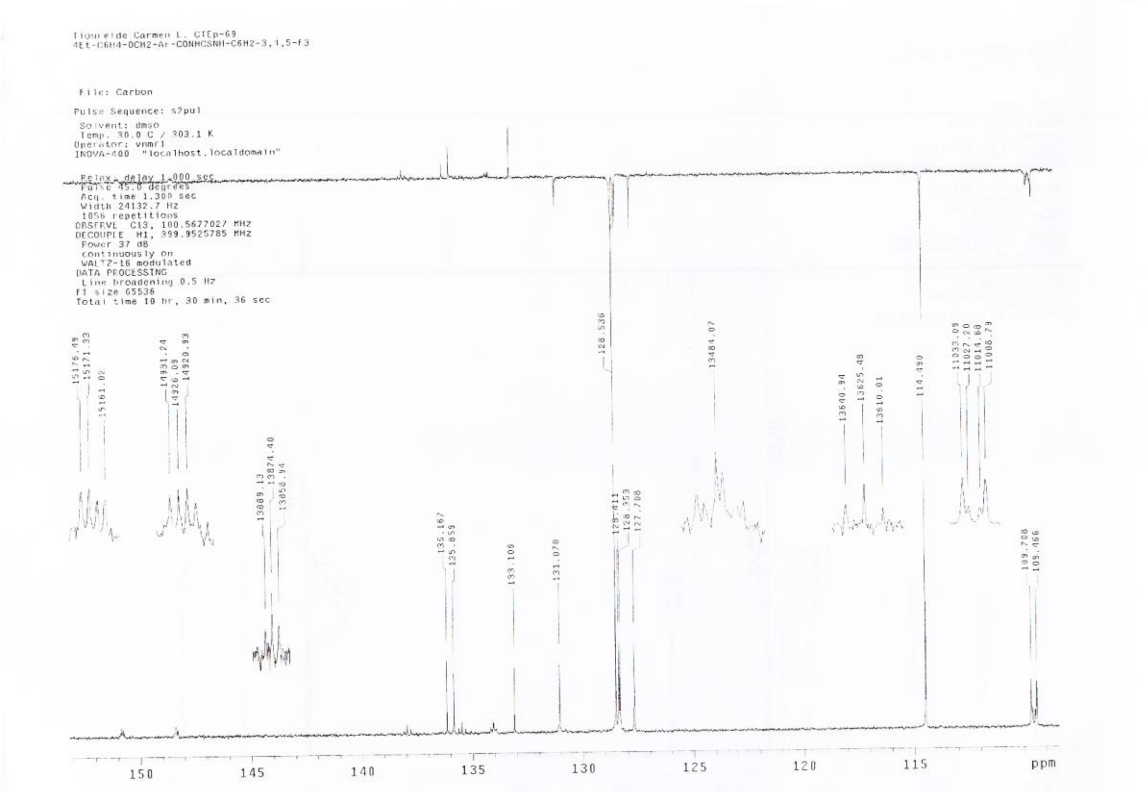

Figure S9. The ^{13}C-NMR spectrum of 2-((4-Eththylphenoxy)methyl)-N-(3,4,5-trifluorophenylcarbamothioyl)benzamide (**5e**).

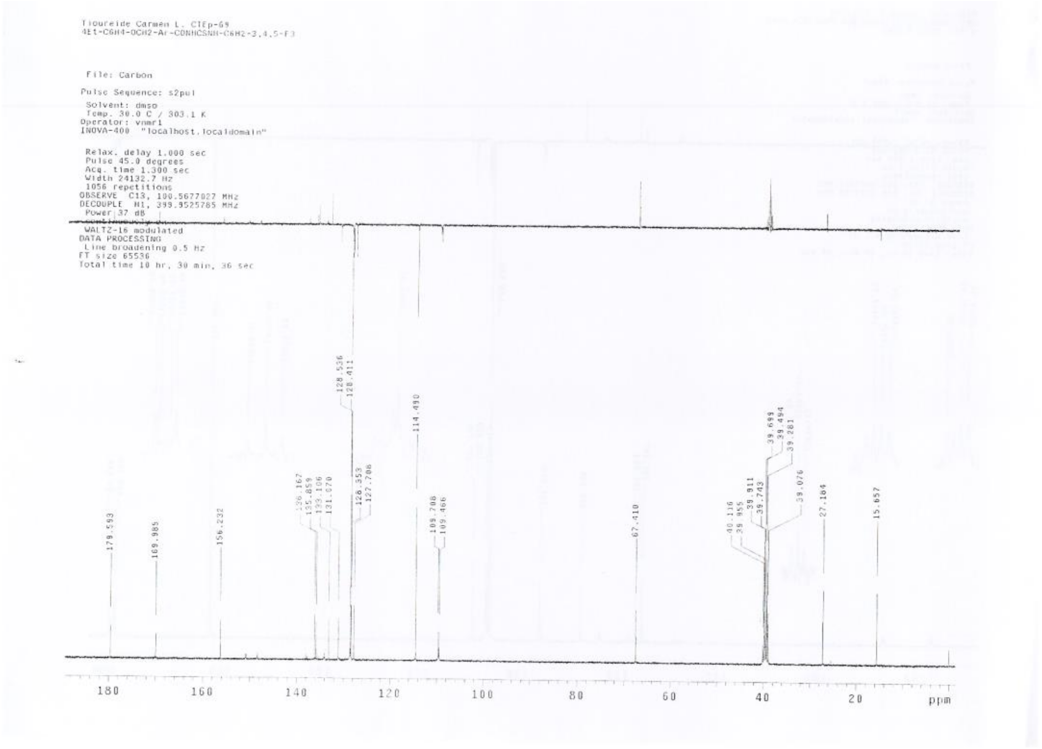

Figure S9. The ^{13}C-NMR spectrum of 2-((4-Ethylphenoxy)methyl)-N-(3,4,5-trifluorophenylcarbamothioyl)benzamide (**5e**).

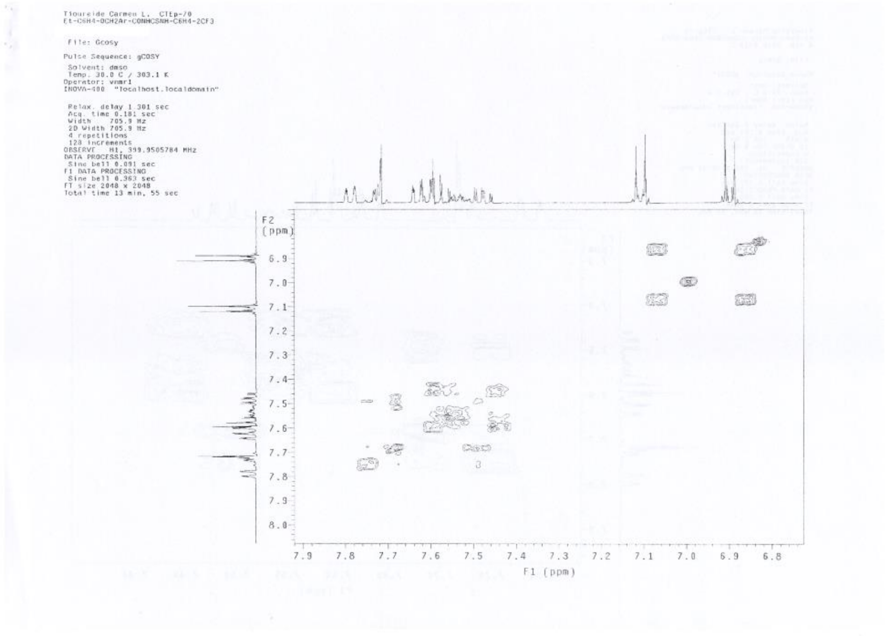

Figure S9. The gCOSY spectrum of 2-((4-Ethylphenoxy)methyl)-N-(3,4,5-trifluorophenylcarbamothioyl)benzamide (**5e**).

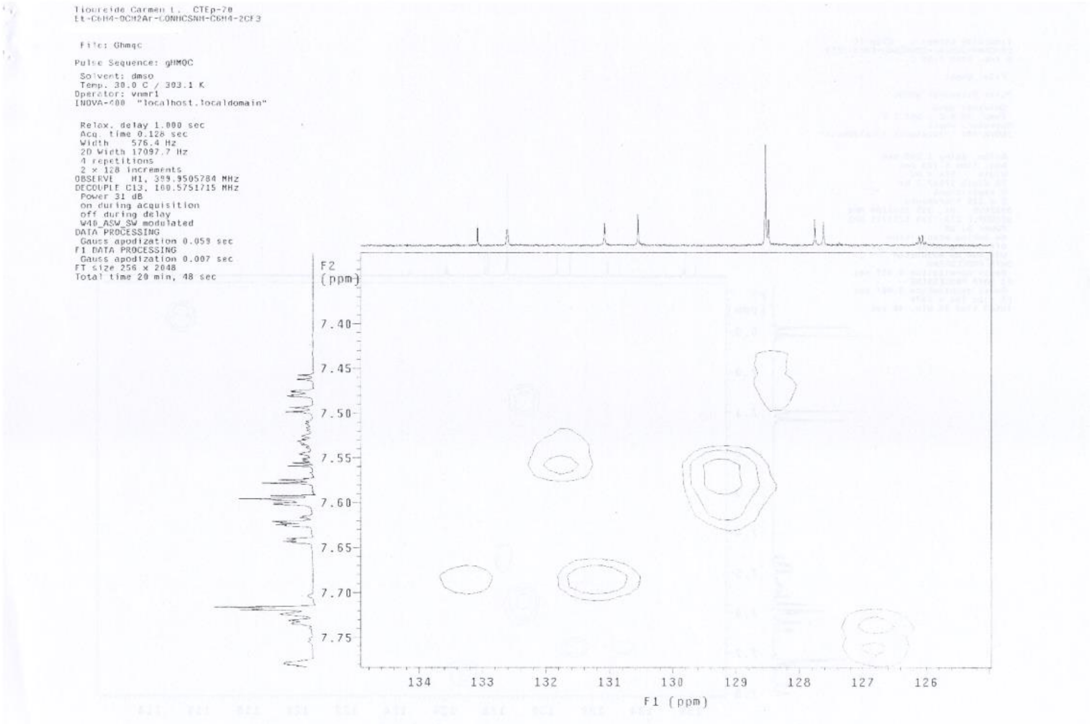

Figure S9. The gHMQC spectrum of 2-((4-Ethylphenoxy)methyl)-N-(3,4,5-trifluorophenylcarbamothioyl)benzamide (**5e**).

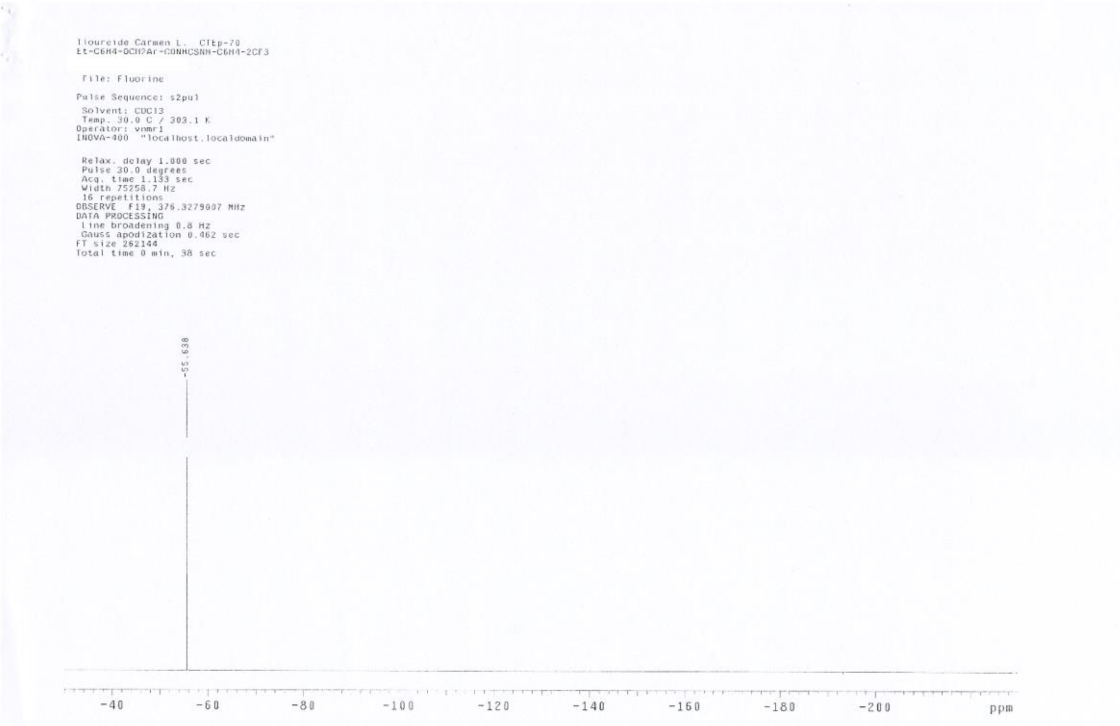

Figure S9. The ^{19}F NMR spectrum of 2-((4-Ethylphenoxy)methyl)-N-(3,4,5-trifluorophenylcarbamothioyl)benzamide (**5e**).

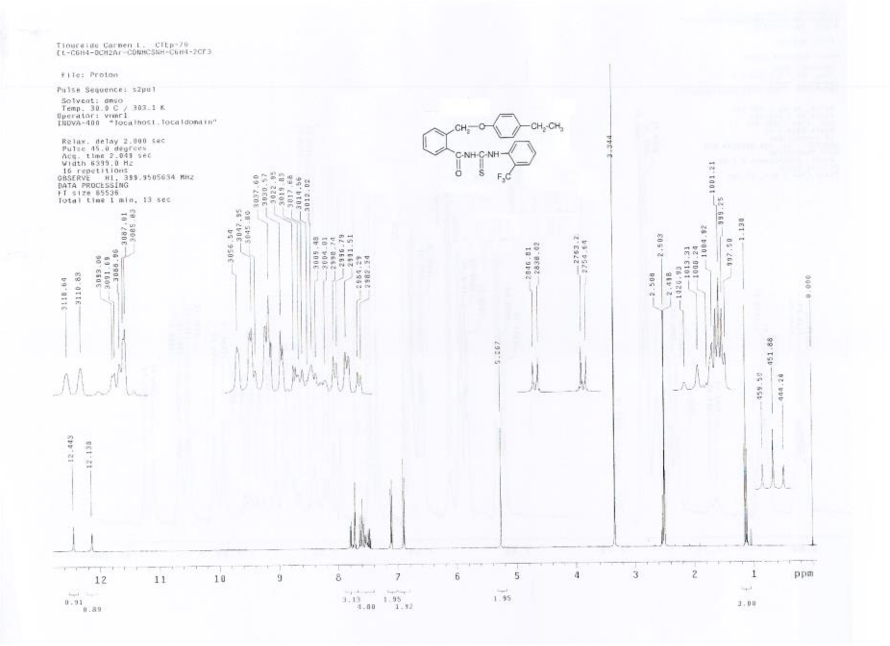

Figure S10. The ^{1}H-NMR spectrum of 2-((4-Ethylphenoxy)methyl)-N-(2-trifluoromethylphenylcarbamothioyl)benzamide (**5f**).

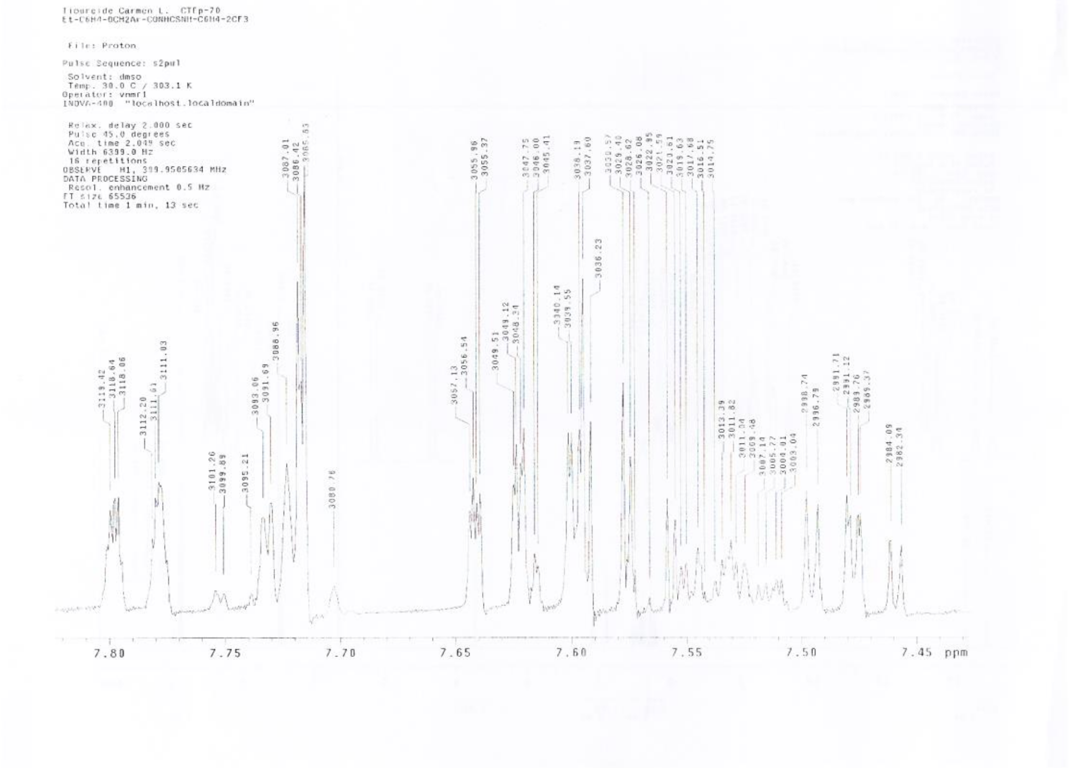

Figure S10. The ^{1}H-NMR spectrum of 2-((4-Ethylphenoxy)methyl)-N-(2-trifluoromethylphenylcarbamothioyl)benzamide (**5f**).

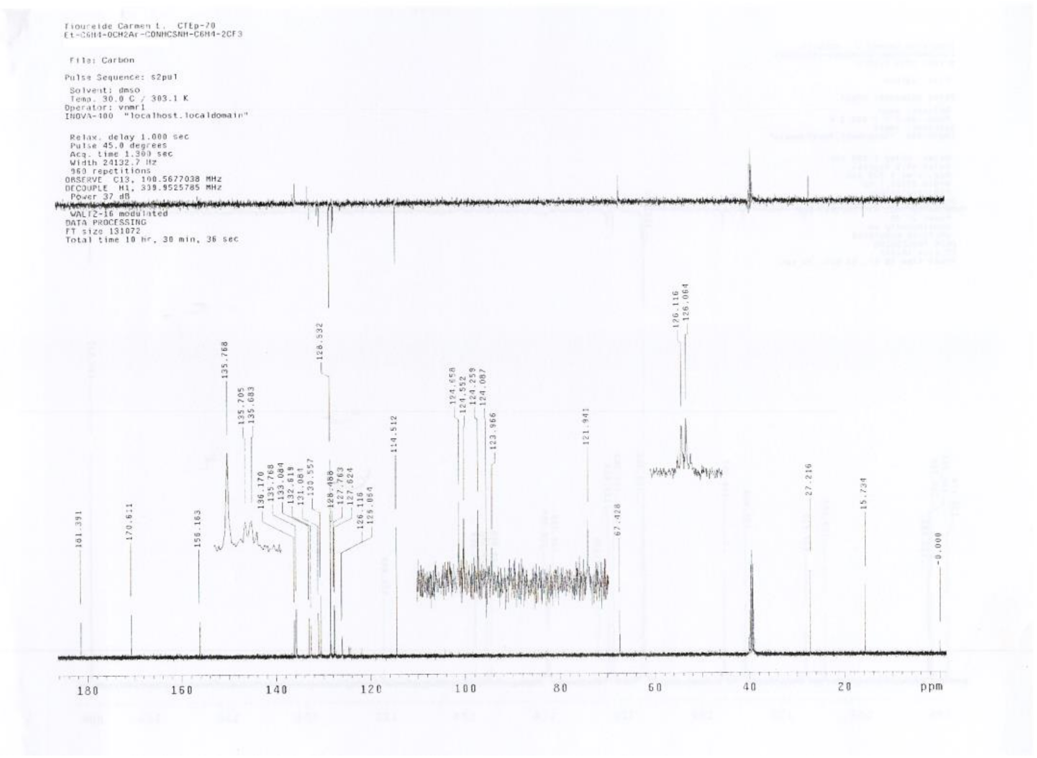

Figure S10. The ^{13}C-NMR spectrum of 2-((4-Ethylphenoxy)methyl)-N-(2-trifluoromethylphenylcarbamothioyl)benzamide (**5f**).

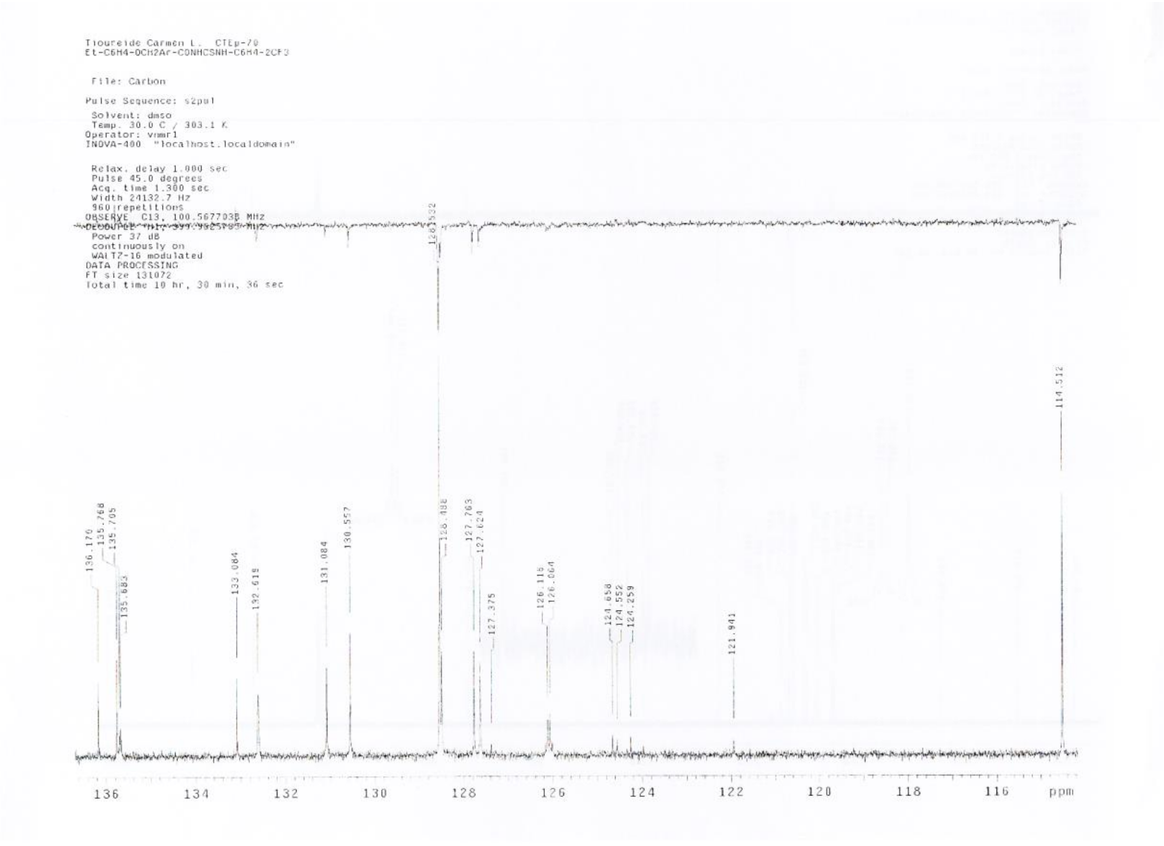

Figure S10. The ^{13}C-NMR spectrum of 2-((4-Ethylphenoxy)methyl)-N-(2-trifluoromethylphenylcarbamothioyl)benzamide (**5f**).

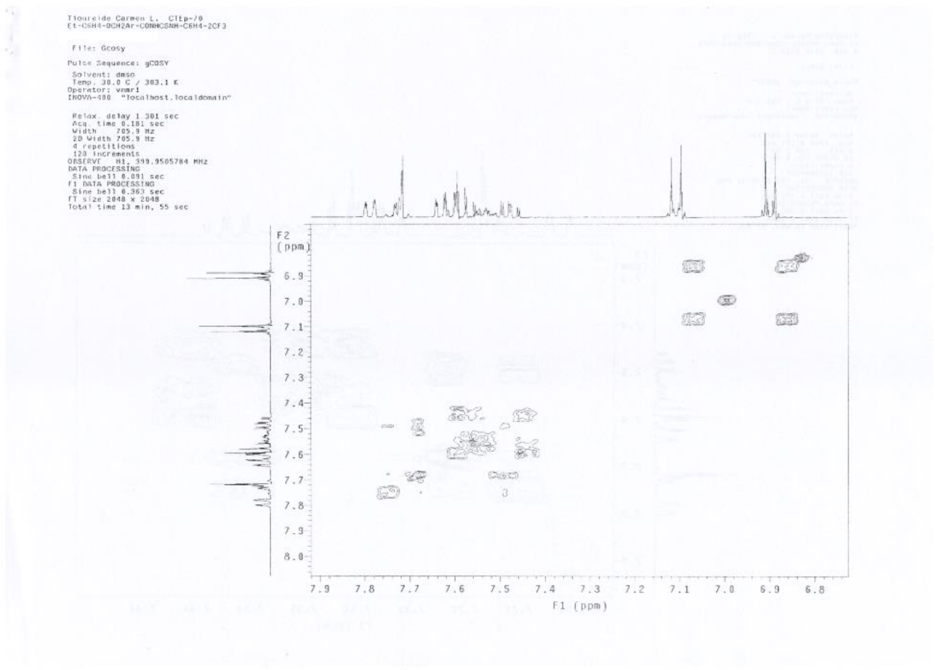

Figure S10. The gCOSY spectrum of 2-((4-Ethylphenoxy)methyl)-N-(2-trifluoromethylphenylcarbamothioyl)benzamide (**5f**).

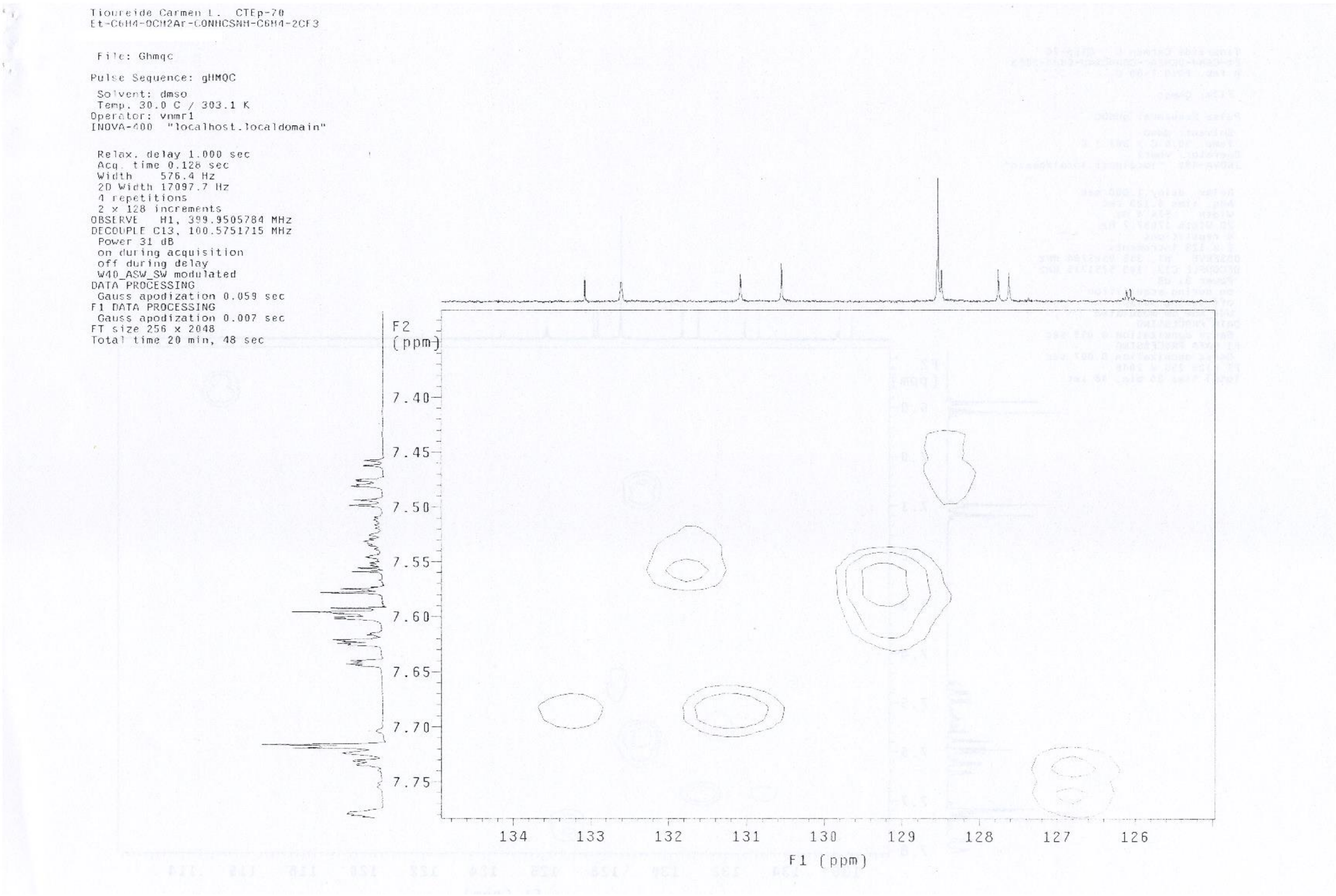

Figure S10. The gHMQC spectrum of 2-((4-Ethylphenoxy)methyl)-N-(2-trifluoromethylphenylcarbamothioyl)benzamide (**5f**).

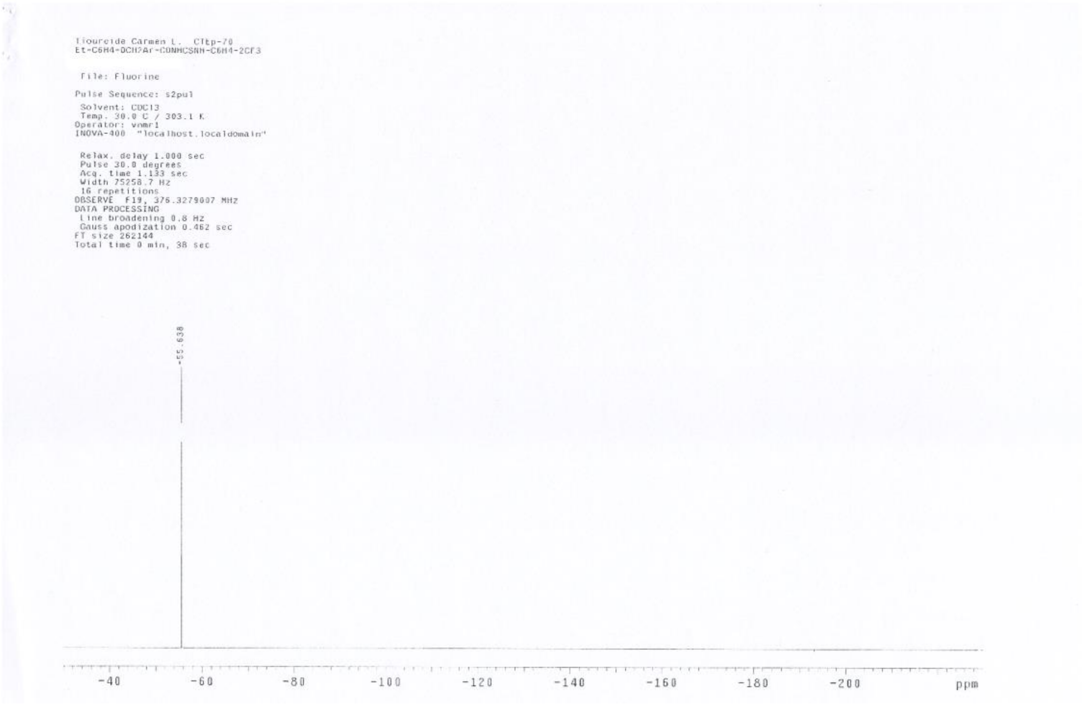

Figure S10. The ^{19}F NMR spectrum of 2-((4-Ethylphenoxy)methyl)-N-(2-trifluoromethylphenylcarbamothioyl)benzamide (**5f**).

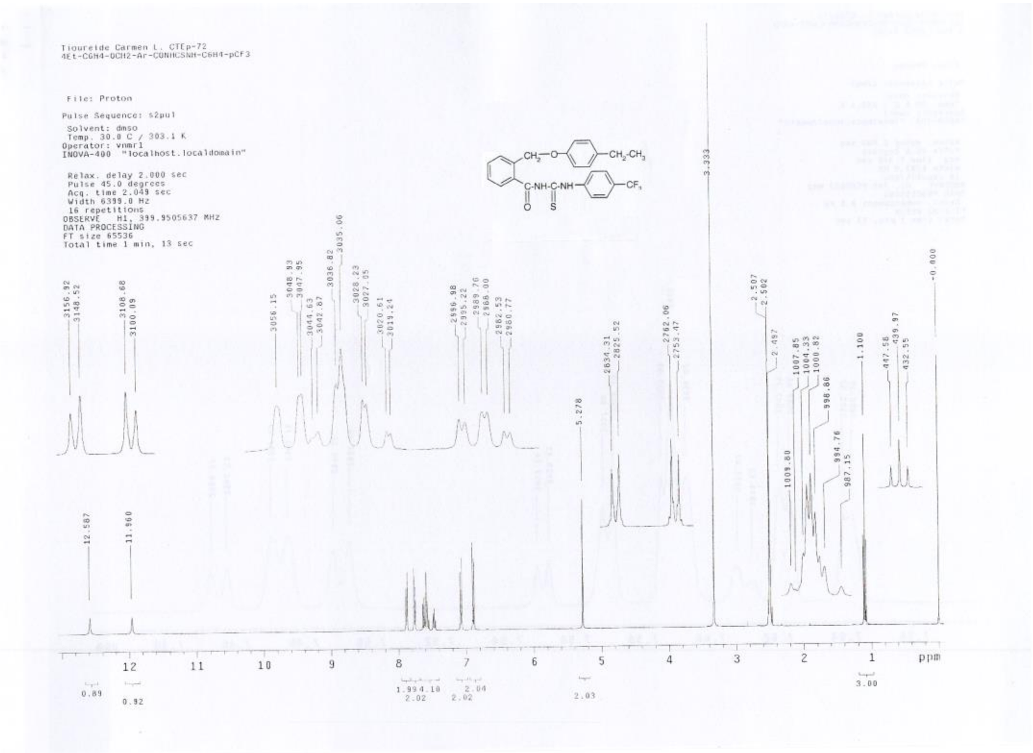

Figure S11. The ^{1}H-NMR spectrum of 2-((4-Ethylphenoxy)methyl)-N-(4-trifluoromethylphenylcarbamothioyl)benzamide (**5g**).

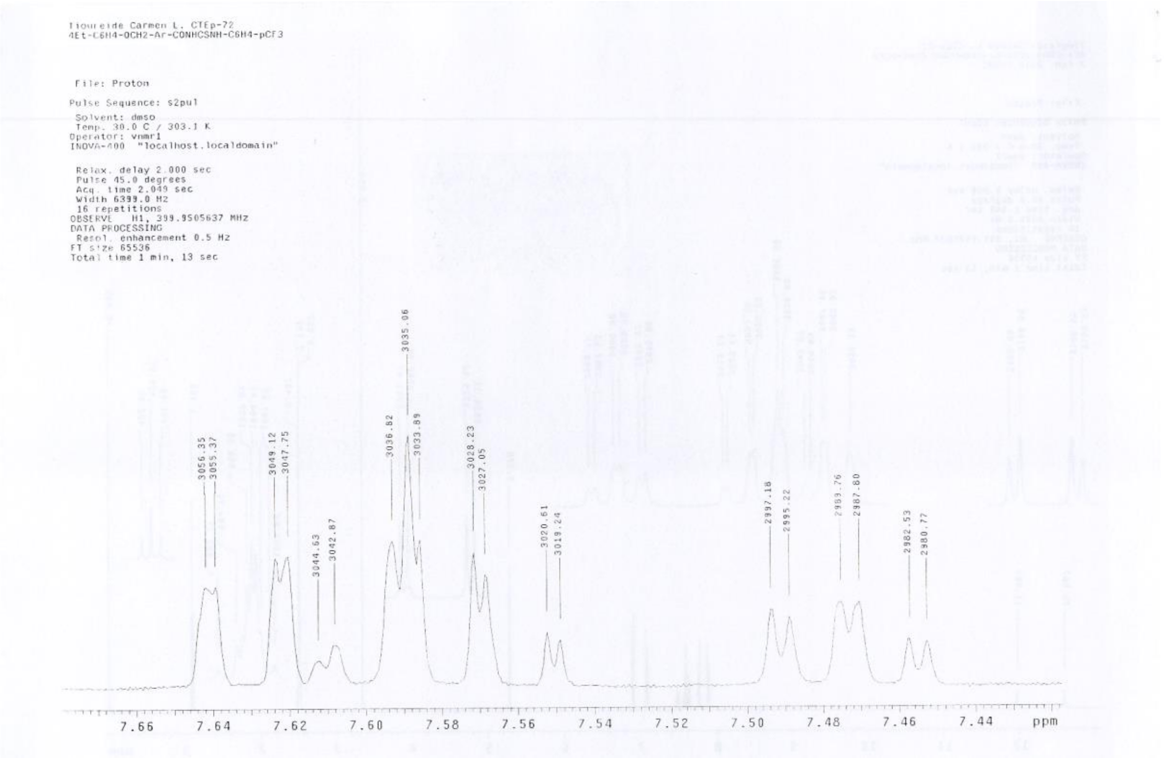

Figure S11. The ^{1}H-NMR spectrum of 2-((4-Ethylphenoxy)methyl)-N-(4-trifluoromethylphenylcarbamothioyl)benzamide (**5g**).

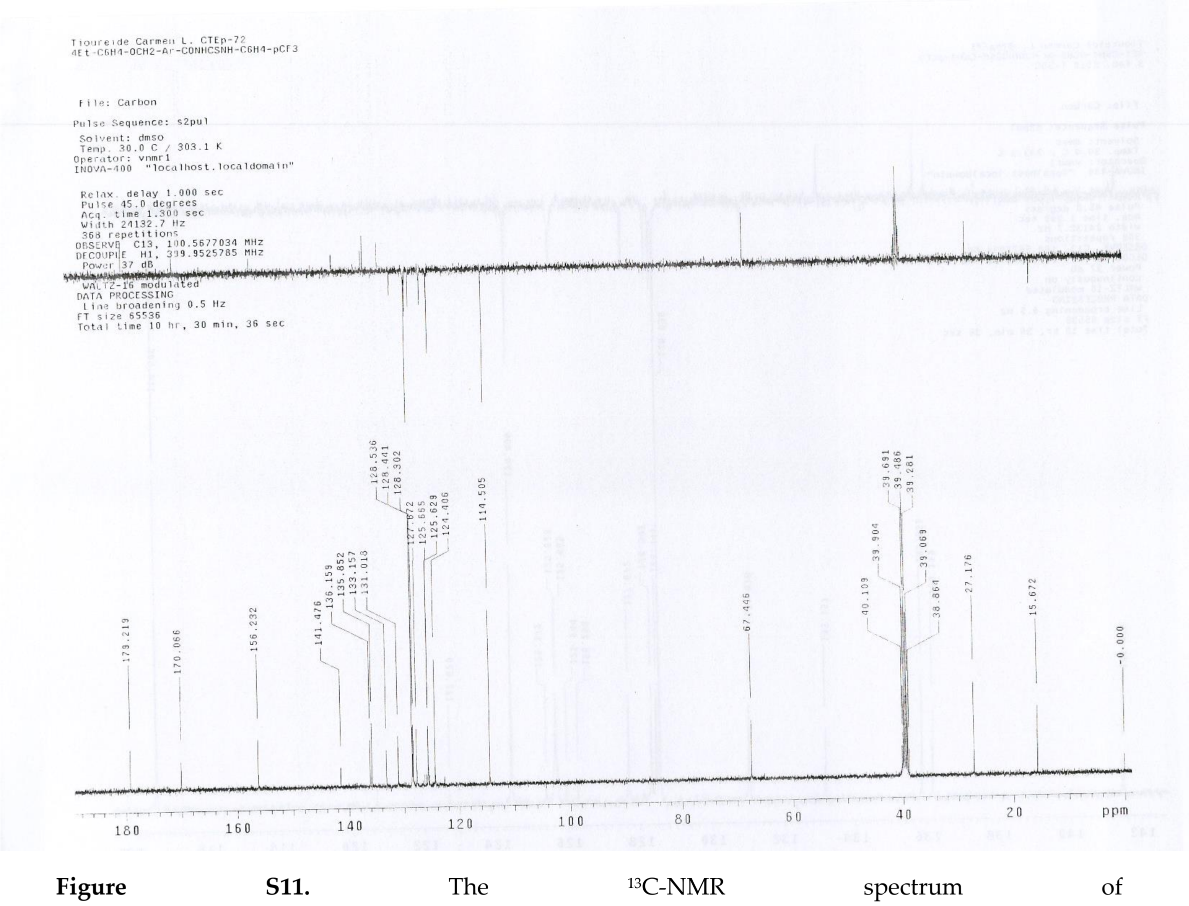

Figure S11. The ^{13}C-NMR spectrum of 2-((4-Ethylphenoxy)methyl)-N-(4-trifluoromethylphenylcarbamothioyl)benzamide (**5g**).

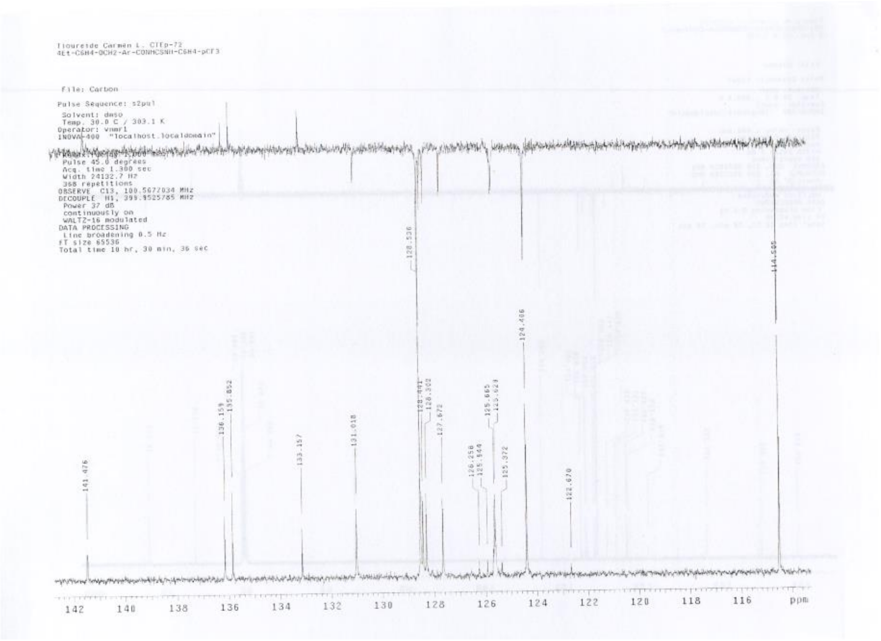

Figure S11. The ^{13}C-NMR spectrum of 2-((4-Ethylphenoxy)methyl)-N-(4-trifluoromethylphenylcarbamothioyl)benzamide (**5g**).

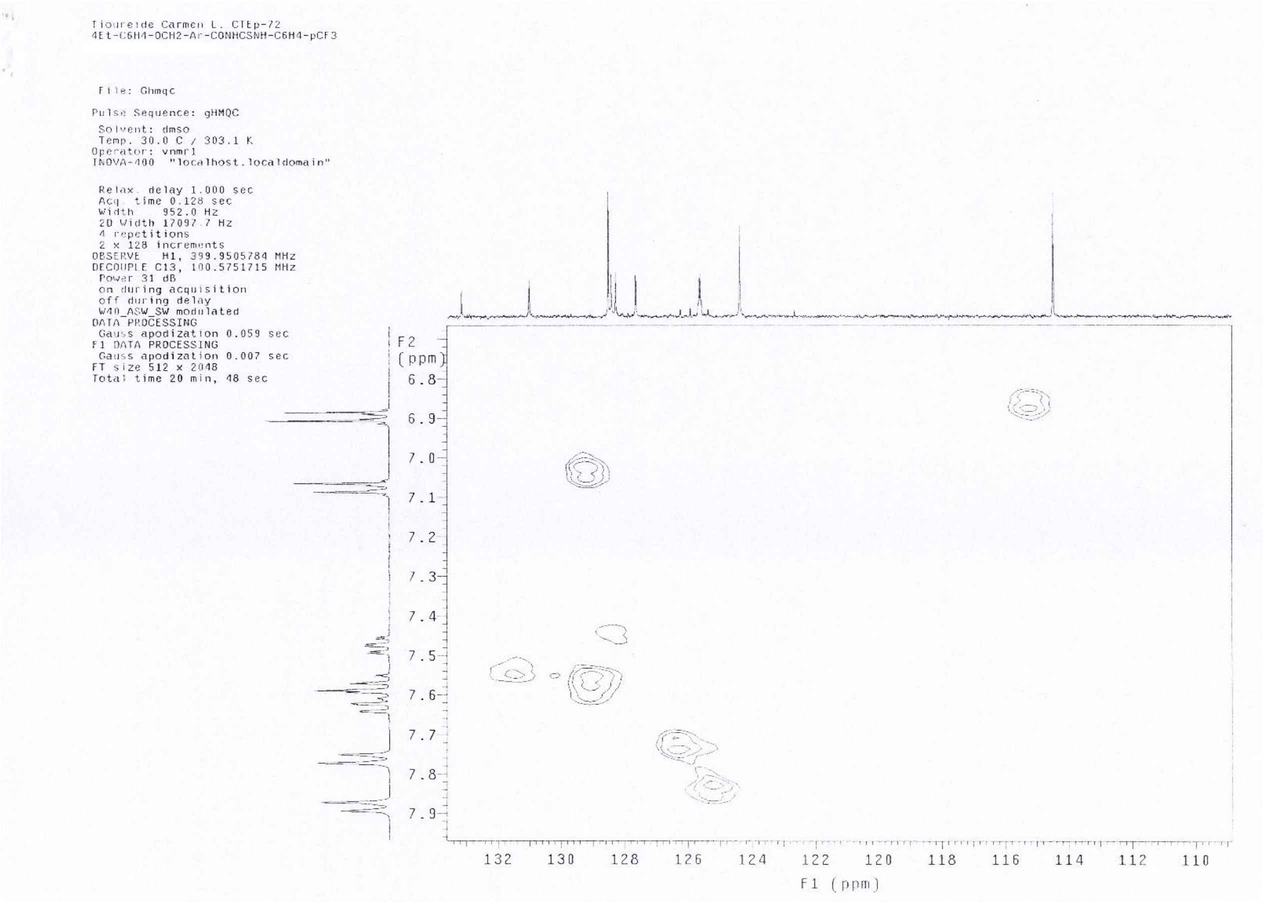

Figure S11. The gHMQC spectrum of 2-((4-Ethylphenoxy)methyl)-N-(4-trifluoromethylphenylcarbamothioyl)benzamide (**5g**).

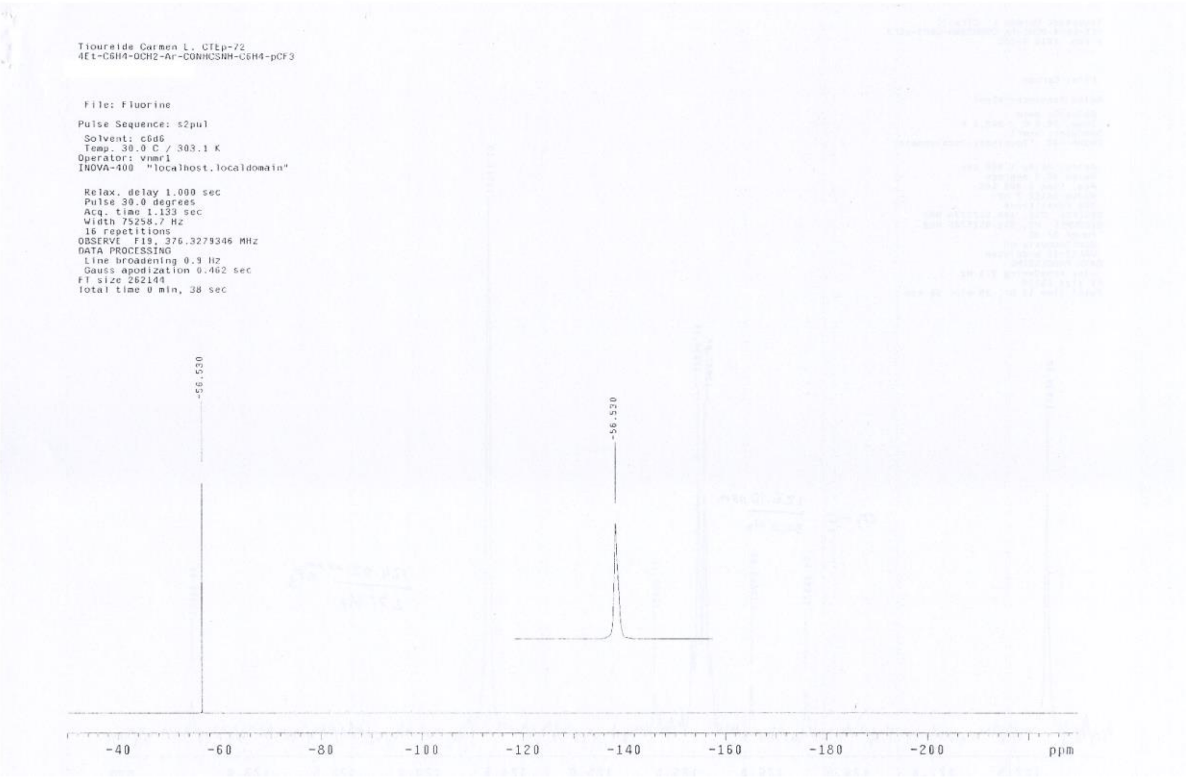

Figure S11. The ^{19}F NMR spectrum of 2-((4-Ethylphenoxy)methyl)-N-(4-trifluoromethylphenylcarbamothioyl)benzamide (**5g**).

Table S1. Crystallographic data for **5d.**

**Compound 5d**	
Empirical formula	$C_{23}H_{19}F_3N_2O_2S$
M	444.47
T(K)	293
λ(nm)	0.71075
Crystal size (mm)	0.400 X 0.350 X 0.200
Crystal color	colourless
Crystal system	Triclinic
Space group	P-1 (#2)
a (Å)	7.1369(2)
b (Å)	11.8876(3)
c (Å)	13.4640(9)
α (°)	102.869(7)
β (°)	91.557(6)
γ (°)	104.241(7)
V (Å^3)	1075.31(10)
Z	2
Calculated density (g.cm^{-3})	1.373
μ (MoK$_\alpha$) (cm^{-1})	1.983
F(000)	460.00
Reflections collected/unique	15071/4927 (R_{int} = 0.0251)
No. observations (All reflections)	4927
No. variables	288
Goodness-of-fit	1.133
Final *R* indices [I>2σ]	0.0435
R indices (all data) *R*1	0.0776
*wR*2	0.1530
Largest diff. peak and hole (e-/Å^{-3})	0.29 and -0.36

Table S2. Selected bond lengths (Å) for **5d.**

**atom.**	**atom**	**distance**	**atom**	**atom**	**distance**
S1	C17	1.653(2)	F1	C23	1.345(2)
F2	C21	1.357(4)	F3	C19	1.344(3)
O1	C6	1.383(3)	O1	C9	1.434(3)
O2	C16	1.220(2)	N1	C16	1.375(3)
N1	C17	1.385(3)	N2	C17	1.339(2)
N2	C18	1.419(3)	C1	C2	1.473(5)
C2	C3	1.537(4)	C3	C4	1.384(3)
C3	C8	1.387(4)	C4	C5	1.387(4)
C5	C6	1.381(3)	C6	C7	1.379(3)
C7	C8	1.396(4)	C9	C10	1.498(3)
C10	C11	1.398(3)	C10	C15	1.385(4)
C11	C12	1.393(3)	C11	C16	1.496(3)
C12	C13	1.386(4)	C13	C14	1.374(5)
C14	C15	1.374(4)	C18	C19	1.380(3)
C18	C23	1.370(3)	C19	C20	1.368(4)
C20	C21	1.362(4)	C21	C22	1.359(4)
C22	C23	1.375(4)			

Table S3. Selected bond lengths (Å) and angles (°) for **5d.**

atom.	atom	atom	angle	atom	atom	atom	angle
C6	O1	C9	116.91(15)	C16	N1	C17	128.47(16)
C17	N2	C18	123.09(18)	C1	C2	C3	113.2(2)
C2	C3	C4	121.9(3)	C2	C3	C8	121.3(2)
C4	C3	C8	116.8(2)	C3	C4	C5	122.2(3)
C4	C5	C6	119.7(2)	O1	C6	C5	116.02(18)
O1	C6	C7	124.2(2)	C5	C6	C7	119.8(2)
C6	C7	C8	119.3(3)	C3	C8	C7	122.1(2)
O1	C9	C10	108.86(18)	C9	C10	C11	121.5(2)
C9	C10	C15	120.2(2)	C11	C10	C15	118.2(2)
C10	C11	C12	120.2(2)	C10	C11	C16	120.69(18)
C12	C11	C16	119.0(2)	C11	C12	C13	120.1(2)
C12	C13	C14	119.5(3)	C13	C14	C15	120.5(3)
C10	C15	C14	121.4(3)	O2	C16	N1	122.7(2)
O2	C16	C11	123.09(18)	N1	C16	C11	114.22(15)
S1	C17	N1	120.51(13)	S1	C17	N2	124.08(17)
N1	C17	N2	115.41(17)	N2	C18	C19	122.33(19)
N2	C18	C23	121.60(18)	C19	C18	C23	116.0(2)
F3	C19	C18	118.3(2)	F3	C19	C20	118.7(2)
C18	C19	C20	123.1(2)	C19	C20	C21	116.9(2)
F2	C21	C20	117.8(2)	F2	C21	C22	118.2(2)
C20	C21	C22	124.0(3)	C21	C22	C23	116.2(2)
F1	C23	C18	117.8(2)	F1	C23	C22	118.38(18)
C18	C23	C22	123.8(2)				

Table S4. Selected bond lengths (Å) for 5d.

Experimental data **X-Ray**			*Calculated data* **B3LYP/6-31G***		
**atom**	**atom**	**distance**	**atom**	**atom**	**distance**
S1	C17	1.653(2)	S1	C17	1.664
F1	C23	1.345(2)	F1	C23	1.346
F2	C21	1.357(4)	F2	C21	1.344
F3	C19	1.344(3)	F3	C19	1.34
O1	C6	1.383(3)	O1	C6	1.38
O1	C9	1.434(3)	O1	C7	1.438
O2	C16	1.220(2)	O2	C16	1.22
N1	C16	1.375(3)	N1	C16	1.398
N1	C17	1.385(3)	N1	C17	1.396
N2	C17	1.339(2)	N2	C17	1.363
N2	C18	1.419(3)	N2	C18	1.41
C1	C2	1.473(5)	C23	C22	1.539
C2	C3	1.537(4)	C2	C3	1.514
C3	C4	1.384(3)	C3	C4	1.404
C3	C8	1.387(4)	C3	C8	1.397
C4	C5	1.387(4)	C4	C5	1.404
C5	C6	1.381(3)	C5	C6	1.4
C6	C7	1.379(3)	C6	C7	1.366
C7	C8	1.396(4)	C7	C8	1.398
C9	C10	1.498(3)	C9	C10	1.507
C10	C11	1.398(3)	C10	C11	1.407
C10	C15	1.385(4)	C10	C15	1.396
C11	C12	1.393(3)	C11	C12	1.398
C11	C16	1.496(3)	C11	C16	1.506
C12	C13	1.386(4)	C12	C13	1.395

C13	C14	1.374(5)	C13	C14	1.394
C14	C15	1.374(4)	C13	C14	1.397
C18	C19	1.380(3)	C18	C19	1.401
C18	C23	1.370(3)	C18	C23	1.398
C19	C20	1.368(4)	C19	C20	1.387
C20	C21	1.362(4)	C20	C21	1.391
C21	C22	1.359(4)	C21	C22	1.39
C22	C23	1.375(4)	C22	C23	1.388
**atom**	atom	distance	**atom**	atom	distance

Table S5. Selected angles (°) for **5d.**

Experimental data X-Ray				Calculated data B3LYP/6-31G*			
atom	atom	atom	angle	atom	atom	atom	angle
C6	O1	C9	116.91(15)	C6	O1	C9	118.11
C16	N1	C17	128.47(16)	C16	N1	C17	131.45
C17	N2	C18	123.09(18)	C17	N2	C18	124.31
C1	C2	C3	113.2(2)	C1	C2	C3	112.87
C2	C3	C4	121.9(3)	C2	C3	C4	121.47
C2	C3	C8	121.3(2)	C2	C3	C8	121
C4	C3	C8	116.8(2)	C4	C3	C8	117.51
C3	C4	C5	122.2(3)	C3	C4	C5	121.93
C4	C5	C6	119.7(2)	C4	C5	C6	119.31
O1	C6	C5	116.02(18)	O1	C6	C5	116
O1	C6	C7	124.2(2)	O1	C6	C7	124.15
C5	C6	C7	119.8(2)	C5	C6	C7	119.85
C3	C8	C7	122.1(2)	C3	C8	C7	121.93
O1	C9	C10	108.86(18)	O1	C9	C10	107.16
C9	C10	C11	121.5(2)	C9	C10	C11	120.1
C9	C10	C15	120.2(2)	C9	C10	C15	120.83
C11	C10	C15	118.2(2)	C11	C10	C15	119
C10	C11	C12	120.2(2)	C10	C11	C12	120.12
C10	C11	C16	120.69(18)	C10	C11	C16	120.4
C12	C11	C16	119.0(2)	C12	C11	C16	119.29
C11	C12	C13	120.1(2)	C11	C12	C13	120.24
C12	C13	C14	119.5(3)	C12	C13	C14	119.89
C13	C14	C15	120.5(3)	C13	C14	C15	119.86
C10	C15	C14	121.4(3)	C10	C15	C14	120.86
O2	C16	N1	122.7(2)	O2	C16	N1	119.59
O2	C16	C11	123.09(18)	O2	C16	C11	121.73
N1	C16	C11	114.22(15)	N1	C16	C11	118.46
S1	C17	N1	120.51(13)	S1	C17	N1	120.67
S1	C17	N2	124.08(17)	S1	C17	N2	125.78
N1	C17	N2	115.41(17)	N1	C17	N2	113.52
N2	C18	C19	122.33(19)	N2	C18	C19	122.26
N2	C18	C23	121.60(18)	N2	C18	C23	121.16
C19	C18	C23	116.0(2)	C19	C18	C23	116.51
F3	C19	C18	118.3(2)	F3	C19	C18	118.51
F3	C19	C20	118.7(2)	F3	C19	C20	118.79
C18	C19	C20	123.1(2)	C18	C19	C20	122.7
C19	C20	C21	116.9(2)	C19	C20	C21	117.67
F2	C21	C20	117.8(2)	F2	C21	C20	118.7

F2	C21	C22	118.2(2)	F2	C21	C22	118.68
C20	C21	C22	124.0(3)	C20	C21	C22	122.62
C21	C22	C23	116.2(2)	C21	C22	C23	117.28
F1	C23	C18	117.8(2)	F1	C23	C18	118.16
F1	C23	C22	118.38(18)	F1	C23	C22	118.64
C18	C23	C22	123.8(2)	C18	C23	C22	123.2

Table S6. Experimental and theoretical ^{1}H-, ^{13}C- and ^{19}F correlation NMR for **5d.**

Experimental data **^{1}H-NMR(dmso-d6, δ ppm, *J* Hz)**	*Calculated data* **DFT/B3LYP/6-31G***
12.14 (br s, 1H, NH)	7.29 (1H, NH)
11.56 (br s, 1H, NH)	6.94 (1H, NH)
7.64 (dd, *J* = 1.2 Hz, *J* = 7.6 Hz, 1H, H-7)	7.54 (^{3}J = 6.9, 1H, H-7)
7.60 (m, 1H, H-4)	7.11 (^{3}J = 6.9, 1H, H-4)
7.57 (td, *J* = 1.2 Hz, *J* = 7.6 Hz, 1H, H-5)	7.24(^{3}J = 6.7 6.9, 1H, H-5)
7.47 (td, *J* = 1.42 Hz, *J* = 7.5 Hz, 1H, H-6)	7.38 (^{3}J = 6.7 6.9, 1H, H-6)
7.30 (m, 2H, H-19, H-21)	6.09(2H, H-19, H-21)
7.08 (d, *J* = 8.6 Hz, 2H, H-11, H-13)	7.06(^{3}J = 7.7, 2H, H-11, H-13)
6.89 (d, *J* = 8.6 Hz, 2H, H-10, H-14)	6.73(^{3}J = 7.7, 2H, H-10, H-14)
5.27 (s, 2H, H-8)	4.89 (2H, H-8)
2.51 (q, *J* = 7.5 Hz, 2H, H-15)	2.53(^{3}J = 6.6(x3), 2H, H-15)
1.14 (t, *J* = 7.5 Hz, 3H, H-15′)	1.18 (^{3}J = 6.6(x2), 3H, H-15′)
Experimental data **^{13}C-NMR (dmso-d6, δ ppm)**	*Calculated data* **DFT/B3LYP/6-31G***
182.27 (C-16)	186.63 (C-16)
169.75 (C-1)	170.31(C-1)
160.82 (dt,$^{3}J_{(C20-F18,22)}$= 30.2 Hz $J_{(C20-F20)}$= 247.1 Hz, C-20)	159.93 (C-20)
158.15 (ddd, $J_{(C-F)}$ = 7.3 Hz, $J_{(C-F)}$ = 16.1 Hz, $J_{(C-F)}$ = 250.0 Hz, C-18, C-22)	158.34 (C-18, C-22)
156.12 (C-9)	155.85 (C-9)
136.19 (Cq)	137.27 (C-2)
135.86 (Cq)	137.15 (C-12)
132.97 (Cq)	135.35 (C-3)
131.04 (C-5)	129.42 (C-5)
128.61 (C-4)	130.06 (C-4)
128.51 (C-11, C-13)	129.54 (C-11, C-13)
128.46 (C-7)	128.35 (C-7)
127.68 (C-6)	129.31 (C-6)
114.68 C-10, C-14)	113.78 (C-10, C-14)
113.35 (td, $J_{(C17-F20)}$ = 5.1 Hz, $J_{(C17-F18, 22)}$ = 16.5 Hz, C-17)	115.47 (C-17)
100.76 (td, $J_{(C19, 21-F20)}$ = 3.6 Hz, $J_{(C19, 21-F18, 22)}$ = 27.1 Hz, C-19, C-21)	100.74 (C-19, C-21)
67.53 (C-8)	68.79 (C-8)
27.21 (C-15)	30.99 (C-15)
15.74 (C-15′)	20.05 (C-15′)
Experimental data **^{19}F-NMR (dmso-d6, δ ppm)**	*Calculated data* **DFT/B3LYP/6-31G***
–108.85 (m, F-20)	–112.14 (F-20)
–114.85 (m, F-18, F-22)	–117.00 (F-18, F-22)

Table S7. The list of intermolecular interactions between the ligand molecules docked with 4DUH (*E. coli* DNA gyrase B) using CLC Drug Discovery Workbench Software.

Comp.	Score/ RMSD	Interacting group	Hydrogen bond	Bond length
**Co-crys-tallized**	−72.04 /0.08	LYS 103:A, ALA 100:A, GLY 102:A, GLY 101:A, ILE 94:A, PRO 79:A, ILE 78:A, ARG 76:A, GLY 75:A, THR 165:A, ASP 73:A, GLN 72:A, VAL 71:A, VAL 167:A, GLU 50:A, ASN 46:A, VAL 120:A, VAL 43:A, ALA 47:A	O sp^2(O12) – N sp^2 from ARG 136:A	3.069 Å
			O sp^2 (O12) – N sp^2 from ARG 136:A	2.700 Å
			O sp^2(O12) – N sp^2 from ARG 76:A	3.138 Å
			N sp^3 (N13) – O sp^2 from GLY 101:A	3.146 Å
			H- O sp^2 from ASP 73:A	1.822 Å
**5a**	−61.58 /0.05	LYS 103:A, ALA 100:A, GLY 102:A, GLY 101:A, ILE 94:A, PRO 79:A, ILE 78:A, ARG 76:A, ARG 136:A, GLY 77:A, MET 166:A, THR 165:A, ASP 73:A, GLN 72:A, VAL 71:A, VAL 167:A, VAL 44:A, GLU 50:A, ASN 46:A, VAL 120:A, VAL 43:A, ALA 47:A, ASP 49:A, ALA 53:A	O sp^3(O1) – N sp^2 from ASN 46:A	3.082 Å
			O sp^2 (O2) – N sp^2 from ILE 78:A	3.305 Å
			N sp^2 (N1) – O sp^2 from GLY 77:A	2.974 Å
**5b**	−59.66 /0.46	LYS 103:A, ALA 100:A, GLY 102:A, GLY 101:A, ILE 94:A, PRO 79:A, ILE 78:A, ARG 76:A, ARG 136:A, GLY 77:A, THR 165:A, ASP 73:A, VAL 120:A, GLY 119:A, SER 121:A, PHE 104:A, HIS 99:A, VAL 118:A, GLY 117:A, GLU 42:A, ASP 45:A, ASN 46:A, ASP 49:A, ASP 73:A	O sp^2(O2) – N sp^2 from LYS 103:A	3.043 Å
			O sp^2 (O2) – N sp^2 from GLY 102:A	3.187 Å
			O sp^2 (O2) – N sp^2 from GLY 101:A	3.119 Å
**5c**	−57.73 /0.08	ASN 46:A, GLU 42:A, GLU 50:A, ARG 76:A, GLY 77:A, ARG 136:A, ILE 78:A, PRO 79:A, ALA 90:A, GLY 102:A, ILE 94:A, MET 95:A, ALA 100:A, LYS 103:A, HIS 99:A, SER 121:A, VAL 120:A, GLY 119:A	N sp^2 (N2) – O sp^2 from GLY 101:A	3.234 Å
**5d**	−56.80 /0.20	GLU 42:A, GLY 119:A, SER 121:A, VAL 120:A, HIS 99:A, ASN 46:A, MET 95:A, ALA 100:A, ILE 94:A, GLY 101:A, ILE 78:A, PRO 79:A, GLY 77:A, ALA 90:A, HIS 83:A, GLY 81:A, ARG 136,:A, ARG 76:A, GLU 50:A, GLY 102: A, LYS 103:A, GLU 42:A, GLY 119:A, HIS 99:A	N sp^2 (N2) – O sp^2 from GLY 101:A	3.144 Å
			N sp^2 (N1) – O sp^2 from GLY 101:A	3.381 Å
**5e**	−63.91 /0.10	VAL 44:A, VAL 71:A, GLN 72:A, ASP 73:A, VAL 167:A, MET 166:A, THR 165:A, ASP 49:A, ASN 46:A, ALA 53:A, VAL 43:A, ALA 47:A, GLU 50:A, LYS 103:A, VAL 120:A, GLY 102:A, GLY 101:A, ALA 100:A, ILE 94:A, PRO 79:A, ILE 78:A, GLY 77:A, ARG136:A	O sp^3(O1) – N sp^2 from ASN 46:A	2.870 Å
			O sp^2 (O2) – N sp^2 from ILE 78:A	3.244 Å
			N sp^2 (N1) – O sp^2 from GLY 77:A	2.924 Å
**5f**	−56.18 /0.22	HIS 83:A, ALA 90:A, ILE 94:A, GLY 101:A, ILE 78:A, PRO 79:A, ARG 136:A, GLY 77:A, ARG 76:A, ARG 164:A, GKY 75:A, THR 165:A, LYS 103:A, VAL 120:A, ASN 46:A, GLU 50:A, ASP 73:A, ALA 47:A	N sp^2 (N2) – O sp^2 from GLY 101:A	2.866 Å
			O sp^3(O1) – N sp^2 from ARG 76:A	3.247 Å
**5g**	−59.92 /0.24	ALA 100:A, VA; 120:A, LYS 103:A, ILE 94:A, GLY 102:A, GLY 101:A, PRO 79:A, ILE 78:A,	N sp^2 (N1) – O sp^2 from GLY 77:A	2.952 Å

	LEU 132:A, GLY 77:A, ARG 136:A, ARG 76:A, GLY 75:A, GLU 50:A, THR 165:A, MET 166:A, ASP 49:A, ASN 46:A, VAL 43:A, VAL 167:A, ALA 47:A, VAL 71:A, ASP 73:A, GLN 72:A	O sp^3(O1) – N sp^2 from ASN 46:A	3.020 Å
